# The Role of Paracrine Regulation of Mesenchymal Stem Cells in the Crosstalk With Macrophages in Musculoskeletal Diseases: A Systematic Review

**DOI:** 10.3389/fbioe.2020.587052

**Published:** 2020-11-26

**Authors:** Hongtao Xu, Chien-Wei Lee, Yu-Fan Wang, Shuting Huang, Lih-Ying Shin, Yu-Hsuan Wang, Zihao Wan, Xiaobo Zhu, Patrick Shu Hang Yung, Oscar Kuang-Sheng Lee

**Affiliations:** ^1^Department of Orthopaedics and Traumatology, Faculty of Medicine, Prince of Wales Hospital, The Chinese University of Hong Kong, Hong Kong, China; ^2^Institute for Tissue Engineering and Regenerative Medicine, The Chinese University of Hong Kong, Hong Kong, China; ^3^Developmental and Regenerative Biology TRP, Faculty of Medicine, School of Biomedical Sciences, The Chinese University of Hong Kong, Hong Kong, China; ^4^Faculty of Medicine, Li Ka Shing Institute of Health Sciences, Prince of Wales Hospital, The Chinese University of Hong Kong, Hong Kong, China; ^5^Department of Orthopadics, China Medical University Hospital, Taichung, Taiwan

**Keywords:** mesenchymal stem cells (MeSH ID D059630), extracellular vesicles (EVs), exosomes, macrophages, musculoskeletal

## Abstract

The phenotypic change of macrophages (Mφs) plays a crucial role in the musculoskeletal homeostasis and repair process. Although mesenchymal stem cells (MSCs) have been shown as a novel approach in tissue regeneration, the therapeutic potential of MSCs mediated by the interaction between MSC-derived paracrine mediators and Mφs remains elusive. This review focused on the elucidation of paracrine crosstalk between MSCs and Mφs during musculoskeletal diseases and injury. The search method was based on the PRISMA (Preferred Reporting Items for Systematic Reviews and Meta-Analyses) and Cochrane Guidelines. The search strategies included MeSH terms and other related terms of MSC-derived mediators and Mφs. Ten studies formed the basis of this review. The current finding suggested that MSC administration promoted proliferation and activation of CD163^+^ or CD206^+^ M2 Mφs in parallel with reduction of proinflammatory cytokines and increase in anti-inflammatory cytokines. During such period, Mφs also induced MSCs into a motile and active phenotype via the influence of proinflammatory cytokines. Such crosstalk between Mφs and MSCs further strengthens the effect of paracrine mediators from MSCs to regulate Mφs phenotypic alteration. In conclusion, MSCs in musculoskeletal system, mediated by the interaction between MSC paracrine and Mφs, have therapeutic potential in musculoskeletal diseases.

## Introduction

The inflammatory processes in response to musculoskeletal diseases and injury, such as bone fractures, osteoarthritis (OA), osteoporosis, tendon injuries, and muscle injuries, are essential for the correct restoration of structure and function to the affected area (Bosurgi et al., 2011; Mianehsaz et al., 2019; Pajarinen et al., 2019; Wang et al., 2019; Yang and Yang, 2019). However, the dysregulation of inflammatory reactions can aggravate the tissue healing results (Saldana et al., 2019).

Macrophages (Mφs) are the critical regulators involved in initiation, propagation, and resolution of inflammatory response throughout the tissue regenerative process. Mφs have a broad spectrum of adaptive phenotypes and functional transitions that might exacerbate and resolve inflammation during tissue repair process (Saldana et al., 2019). In 2008, Mosser and Edwards analyzed the phenotypic changes of Mφs and robustly classified Mφs into two types: M1 and M2 (Mosser and Edwards, 2008). Proinflammatory Mφs are identified as the classic M1 Mφs, which are involved in the early stages of tissue repair, whereas the anti-inflammatory Mφs are identified as M2 Mφs, which dominated later stages of tissue repair (Murray et al., 2014; Spiller and Koh, 2017). Upon injury, the early presence of M1 Mφs initiates tissue repair, but the persistent of M1 activity can deteriorate the repair process (Krzyszczyk et al., 2018). On the other hand, the early presence of M2 can prevent cellular and vascular infiltration that impairs tissue development through ectopic secretion of fibrotic chemokines and cytokine (Stahl et al., 2013; Bility et al., 2014; Moore et al., 2015). Moreover, several different M2 subtypes have been identified, including M2a, M2b, M2c, and M2d (Stein et al., 1992; Donnelly et al., 1999; Anderson and Mosser, 2002). The undisciplined regulation of Mφ phenotypic change impairs tissue repair, and each of the subtypes might have specific functions; therefore, further investigation is needed to identify the explicit role of M2 subtypes (O'Brien et al., 2019).

In the 1980's, Arnold Caplan and his colleagues published an isolation method of fibroblast-like stromal cells from bone marrow and first identified them as mesenchymal stem cells (MSCs) because of their multilineage differentiation potential (Caplan, 1991). A rapid expansion in the field of MSC-based therapy in immunomodulation and regenerative medicine has been acknowledged (Kingery et al., 2019). It has been reported that MSCs can switch M1 Mφs or resting Mφs into M2 Mφs (Kim and Hematti, 2009; Nemeth et al., 2009; Melief et al., 2013b). Previous studies showed that prostaglandin E_2_ (PGE_2_), interleukin-4 (IL-4), IL-6, and IL-10 released from MSCs can induce the Mφ polarization toward M2 to accelerate tissue regeneration (da Costa Goncalves and Paz, 2019). On the other hand, such immunomodulatory ability of MSCs is adjusted by inflammatory factors released by macrophages (Waterman et al., 2010; Carrero et al., 2012). After the stimulation, MSCs would secrete anti-inflammatory factors, such as transforming growth factor β (TGF-β) and CCL-18, to further suppress activation of lymphocytes and inhibit major histocompatibility complex (MHC) class II and CD86 in lipopolysaccharide (LPS)–stimulated Mφs (Melief et al., 2013b; Cho et al., 2014). This suggested that the interplay between MSCs and Mφs is in control of inflammation, and their crosstalk may be recommended to advocate tissue healing or repair.

Besides versatile soluble proteins, MSC-derived extracellular vesicles (MSC-EVs) have raised worldwide attention in regenerative medicine because of their immunomodulation ability (Raposo and Stoorvogel, 2013). EVs represent a heterogeneous group of cell-derived membranous vesicles, such as microvesicles (MVs) and exosomes, which were first described in the 1970's (Vakhshiteh et al., 2019). MVs, ranging from 40 to 2,000 nm in diameter (Bruno et al., 2012; Kim et al., 2012), and exosomes, ranging from 30 to 150 nm in diameter (Li et al., 2013; Zhao et al., 2015), are critical mediators for intercellular communication via the delivery of the embedded RNAs, DNAs, and cytosolic proteins. Moreover, boosting the therapeutic potential of MSC-EVs by changing the intrinsic bioactive factors or modifying membrane by bioengineering approach is possible (Lu and Huang, 2020). Refining the culture condition of MSCs could significantly increase the production yield and improve the efficacy of MSC-EVs (Luan et al., 2017; Willis et al., 2017; Bagno et al., 2018; Cha et al., 2018; Ferguson et al., 2018). Also, MSC-derived EVs could serve as a promising drug delivery vector, owing to their high biocompatibility, high efficacy of delivery, and low immunogenicity (Clayton et al., 2003; Ridder et al., 2014; Zomer et al., 2015). EVs derived from different origins have preferential targeting cells due to their distinct membrane composition gained from their parental cells that impart differential effect on body systems (Luan et al., 2017; Ferguson et al., 2018), which could be addressed by exploiting the nature of EVs as natural carriers of miRNA or other molecules by considering them as drug delivery vehicles (Cheng et al., 2017). In addition, MSC-derived EVs in conjunction with other materials might provide substantial advances in both immunomodulation and tissue regeneration (Cosenza et al., 2018). And it has been reported that a variety of material's environment could affect cell's downstream response by cell–material interactions (Darnell et al., 2018). Taken together, bioengineered MSC-derived EVs are novel adjustable biomaterials for tissue regeneration.

The therapeutic effect of MSCs showed a clinical benefit in children suffering from osteogenesis imperfecta (Otsuru et al., 2012) and preclinical benefit in bone fracture (Li Y. et al., 2019), OA (Cosenza et al., 2017), tendon injury (Chamberlain et al., 2019), pulmonary hypertension (Lee et al., 2012), cancer (Silva et al., 2013), and infectious diseases (Cheng and Schorey, 2013). The mechanisms of these mentioned therapeutic potentials of MSCs need elucidation. This review aimed to expound the role of MSC paracrine, especially MSC-derived EVs, in the crosstalk with Mφs in musculoskeletal diseases. Moreover, a systemic understanding of MSC-EVs properties and activities will provide a solid foundation to boost MSC-EVs for regenerative medicine and will significantly facilitate the translation value of MSC-based therapy (Zhang et al., 2020).

## Methods

The Preferred Reporting Items for Systematic Reviews and Meta-Analyses (PRISMA) statement was used for this article. Meanwhile, the Cochrane handbook was selected as guidelines for the study protocol (Moher et al., 2009).

### Search Strategy and Study Selection

Two different investigators (H.-T.X., L.-Y.S.) conducted the customized up-to-date literature search. PubMed database and EMBASE (Excerpta Medica Database) database were selected in this study.

The title and abstract field were selected to search for MeSH terms and other related terms, which pertained to “Mesenchymal Stem Cells,” “Macrophages,” and “Paracrine Regulation,” such as “Exosomes,” “Extracellular Vesicles” and “Culture Media, Conditioned.” The details of selected search terms and searching procedures that were used in the individual database are available in Appendices 1–3 in Supplementary Material. Additional studies were also located by searching papers referenced in listed articles. Those studies identified by the search outcomes were combined, and duplicates were excluded. Then, the screening procedure of titles and abstracts was performed before elaboration on the selected full-text articles. Two investigators (H.-T.X., L.-Y.S.) screened the titles and abstracts of those identified studies individually. In cases of disagreement between the two authors, a consensus was reached by discussion with a third author (C.-W.L.). After that, the full text of screened articles were examined. Corresponding authors of the reviewed articles could be contacted with essential needs to obtain those missing data.

**Figure 1:**
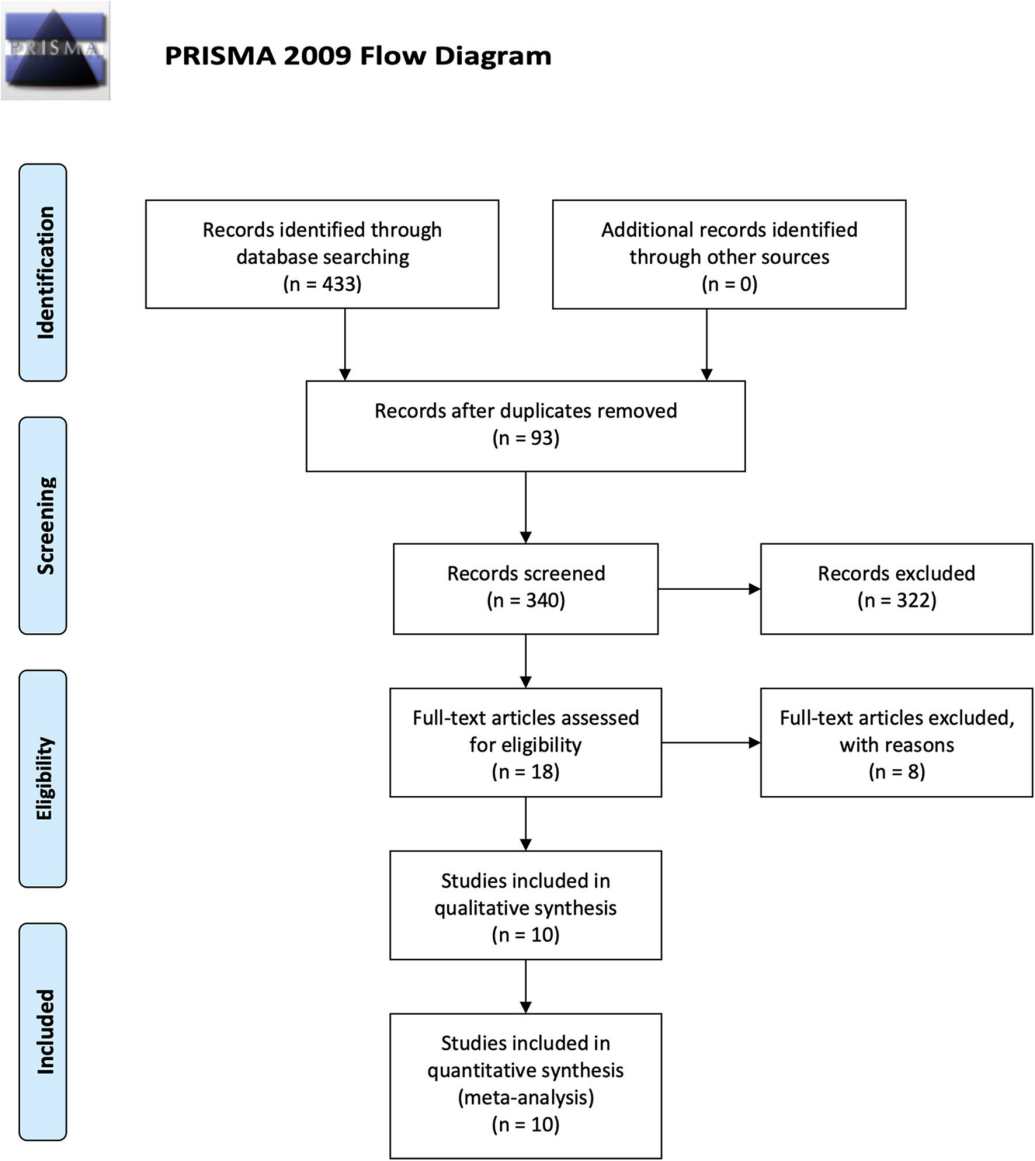
Flowchart presenting the results of the literature search and the strategy used to select studies that relate to the crosstalk between MSCs and Mφs of musculoskeletal diseases. Study selection process. The search revealed 433 records. A total of 93 overlaps were removed between the databases. The remaining 340 records were screened by title and abstract, and 322 records were excluded. The remaining 18 studies were examined using their full texts, and finally ten eligible studies were identified.

The search yielded 433 studies across all databases (239 studies across PubMed database and 194 studies across EMBASE database). A total of 93 duplicates were removed. According to the inclusion and exclusion criteria, all these studies were screened and reviewed by titles and abstracts first; 322 studies were excluded because they were review articles, case reports, case series, letters, chapters, or studies published more than 10 years ago. Of the 18 remaining studies, which were applied by a filter to include musculoskeletal-related studies, eight studies were excluded via reviewing full text. The 10 remaining studies underwent secondary full-text review and were confirmed as fitting the inclusion criteria. The flowchart of the selected studies selection process, which was based on the PRISMA 2009 Flow Diagram (Moher et al., 2009), is shown in Figure 1, and the details of inclusion and exclusion criteria are shown in Table 1.

**Table 1:** Inclusion and exclusion criteria.

**Criteria**	
Inclusion criteria	1. Full-text available;
	2. Written in English;
	3. Articles published in the last 10 years;
	4. Articles containing original data;
	5. Studies must be related to “mesenchymal stem cells” and “macrophages”;
	6. Musculoskeletal related studies.
Exclusion criteria	1. No control group;
	2. Sampling method described inconsistent;
	3. Case reports, case series and review articles, letter, chapter;
	4. Not available in the English language.

### Data Extraction

All relevant data were extracted by H.-T.X. and X.-B.Z.: author information, published journal, year of publication, sample source, target disease, study type (*in vivo, in vitro*, or both), cell management, and measurement instrument. The details of results extraction consisted of variable/control group descriptions, laboratory effects, proposed mechanisms, article conclusions, and research implications. After that, the selected articles were classified according to the type of target disease.

### Methodological Quality Assessment

The methodological assessment should be used as an essential procedure, which could exclude articles with a large degree of bias or with a higher degree of potential bias, has been highlighted to readers (McElvany et al., 2015). Identified studies fitting all criteria were reviewed, and all included data were extracted and analyzed based on study heterogeneity and methodological quality. Because of the natural heterogeneity of measurement across studies, a meta-analysis could not be performed. Two independent authors (H.-T.X. and Z.-H.W.) separately assessed and graded the methodological quality of all selected studies. Disagreements between the two independent researchers were identified and resolved by discussion with a third reviewer (C.-W.L.). The selected studies were assessed with a quality scoring system raised by Wells and Julia (2009) (Appendix 4 in Supplementary Material). The quality assessment system was based on the following eight questions: Was the study hypothesis/aim/objective clearly described? Were the animal models for the study well-described? Were the methods well-described? Were the data collection time point clearly defined? Were the main outcome measures clearly defined? Were the experiment group well-compared with the control group? Were the results well-described? Were the articles discussed the limitation? For each question, 1 point was allocated for “yes,” and 0 point was allocated for “no.” The number of “yes” answers was counted for each selected study to give a total score out of 8. A study's rating was considered as excellent with a score ranging from 6 to 8, good with a score ranging from 4 to 6, poor with a score ranging from 2 to 4, and bad with a score ranging from 0 to 2.

## Results

### Study Methodology Quality Assessment

The scoring system was used to calculate all ten selected studies in Appendix 5 in Supplementary Material. The mean score is 6.6 (range, 5–8), including eight studies exceeding 6 points (Cosenza et al., 2017; Lo Sicco et al., 2017; Hyvarinen et al., 2018; Zhang et al., 2018; Chamberlain et al., 2019; Li Y. et al., 2019; Shi et al., 2019; Shen et al., 2020).

### Study Characteristics

**Figure 2:**
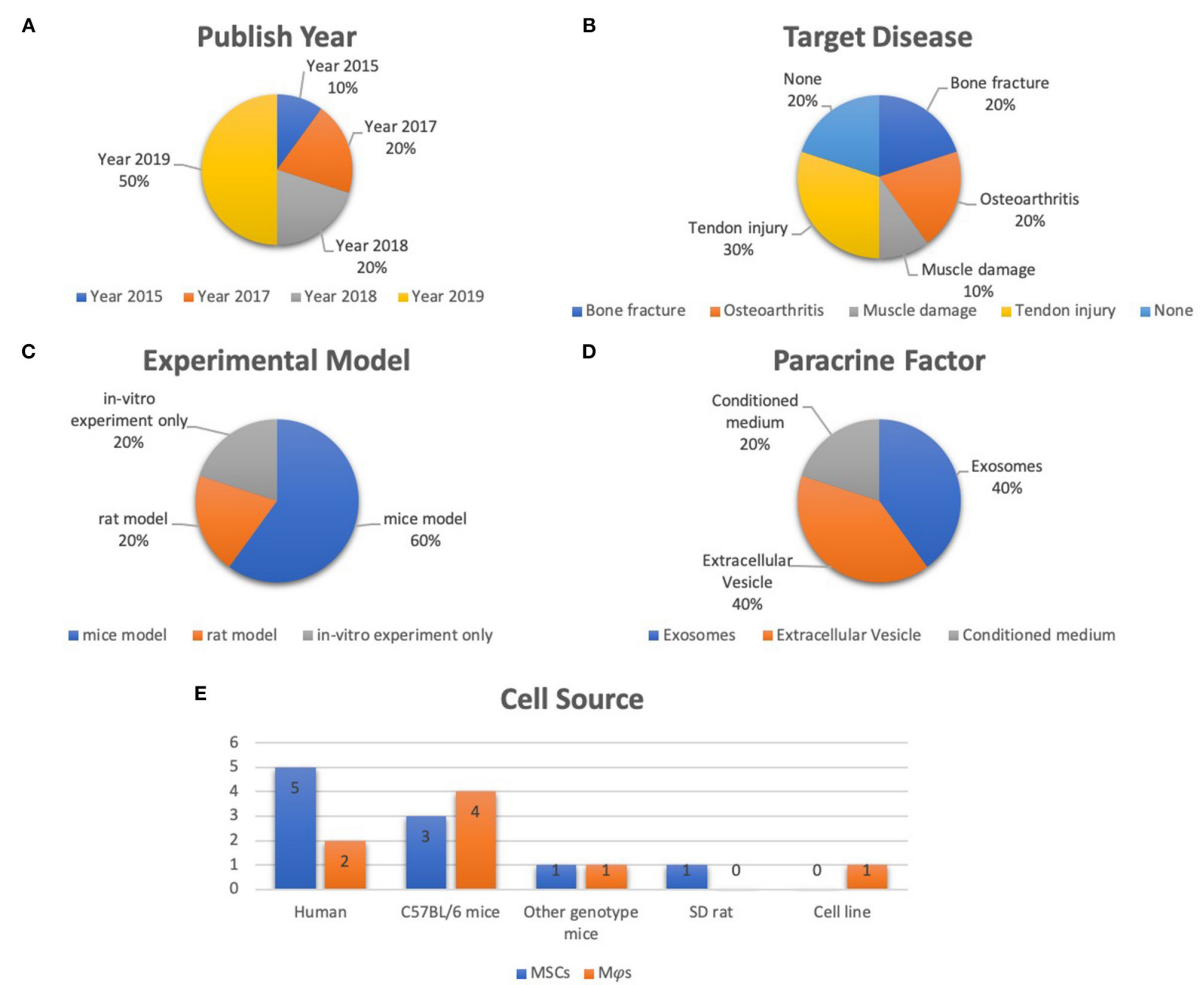
Representative graph of the included studies presented in the articles reviewed. **(A)** Publication year of included studies (range from 2015 to 2020). **(B)** Target disease of included studies (include bone fracture, OA, muscle damage, tendon injury). **(C)** Experimental model of included studies (including mice model, rat model, *in vitro* experiment only). **(D)** Paracrine factors (including extracellular vesicle, exosomes, CM). **(E)** Cell source of included studies (including human, C57Bl/6 mice, other genotype mice, SD rat, cell line).

Of the ten selected studies, one study was published in 2015 (Chang et al., 2015), two were published in 2017 (Cosenza et al., 2017; Lo Sicco et al., 2017), two studies were published in 2018 (Hyvarinen et al., 2018; Zhang et al., 2018), four studies were published in 2019 (Chamberlain et al., 2019; Li Y. et al., 2019; Pacienza et al., 2019; Shi et al., 2019), and the final one was published in 2020 (Shen et al., 2020) (Figure 2A). All selected studies were related to the musculoskeletal system. For details of target diseases, two studies were bone fracture–related studies (Chang et al., 2015; Li Y. et al., 2019), two studies were OA-related studies (Cosenza et al., 2017; Zhang et al., 2018), one study was a muscle damage–related study (Lo Sicco et al., 2017), three studies were tendon injury–related studies (Chamberlain et al., 2019; Shi et al., 2019; Shen et al., 2020), and the other two studies were included without targeting disease (Hyvarinen et al., 2018; Pacienza et al., 2019) (Figure 2B). Six studies used a mice model (Cosenza et al., 2017; Lo Sicco et al., 2017; Chamberlain et al., 2019; Li Y. et al., 2019; Pacienza et al., 2019; Shen et al., 2020), two studies used a rat model (Zhang et al., 2018; Shi et al., 2019), and two studies performed *in vitro* experiments only (Chang et al., 2015; Hyvarinen et al., 2018) (Figure 2C). Three paracrine factors were extracted from the results: MSC-derived exosomes (Cosenza et al., 2017; Zhang et al., 2018; Pacienza et al., 2019; Shen et al., 2020), MSC-derived EVs (Lo Sicco et al., 2017; Hyvarinen et al., 2018; Chamberlain et al., 2019; Shi et al., 2019), and MSC-derived conditioned medium (CM) (Chang et al., 2015; Li Y. et al., 2019) (Figure 2D). Five MSC and Mφs cell sources for EVs isolation were described in these selected studies: human (Lo Sicco et al., 2017; Hyvarinen et al., 2018; Zhang et al., 2018; Chamberlain et al., 2019; Pacienza et al., 2019), cell lines (Pacienza et al., 2019), Sprague–Dawley rats (Shi et al., 2019), C57BL/6 mice (Chang et al., 2015; Cosenza et al., 2017; Lo Sicco et al., 2017; Li Y. et al., 2019), and transgenic mice [scleraxis–green fluorescent protein (GFP) tendon reporter mice, nuclear factor (NF-κB)–GFP–luciferase transgenic reporter mice, and wild-type FVB/NJ (FVB) mice] (Shen et al., 2020). The detailed data are graphically shown in Figure 2E.

### Target Diseases and Experimental Animal Models

In the bone fracture–related studies (Chang et al., 2015; Li Y. et al., 2019), Li Y. et al. (2019) established a cylindrical bone defect mice model by using an electric drill to make a defect with 1-mm diameter and 1-mm depth at the bone callus of the femur. Chang et al. (2015) studied only *in vitro* experiments, and they cocultured bone marrow–derived mesenchymal stem cells (BMSCs) with Mφs. Two studies selected OA as the target disease (Cosenza et al., 2017; Zhang et al., 2018). Cosenza et al. (2017) established a collagenase-induced arthritis mouse model by intra-articularly injecting 1U type VII collagenase (in 5 μL saline) into the knee joint of 10-week-old C57BL/6 mice at days 0 and 2. Zhang et al. (2018) generated an osteochondral defect model in an 8-week-old Sprague–Dawley rat. Osteochondral defects, with 1.5-mm diameter and 1-mm depth, were generated on the trochlear grooves of the distal femurs by a drill bit. For muscle injury study, cardiotoxin-induced muscle injury was applied in 8-week-old male C57BL/6 mice by intramuscular administration of cardiotoxin into the Tibialis Anterior (TA) muscle (Lo Sicco et al., 2017). Three tendon injury models were used in the selected studies (Chamberlain et al., 2019; Shi et al., 2019; Shen et al., 2020). Chamberlain et al. (2019) established a surgically transected Achilles tendon mouse model. After superficial digital flexor tendon was removed, the Achilles tendon was completely transected at the midpoint. Then tendon was sutured together by using 5-0 Vicryl suture. In Shen et al. (2020) study, Achilles tendon was two-thirds transected at the midpoint part between calcaneal insertion and the musculotendinous junction and then was sutured with a two-strand modified Kessler technique. Shi et al. (2019) applied the Sprague–Dawley rat patellar tendon defect model. Briefly, the central one-third of the patellar tendon was removed from the distal apex of the patellar to the insertion of the tibial tuberosity to achieve a tendon structural defect condition. In addition, Pacienza et al. (2019) established a mouse endotoxemia model by injecting LPS via tail vein, which was used to study the crosstalk between MSC-derived exosomes and Mφs. Hyvarinen et al. (2018) only cocultured BMSCs and BMSC-derived EVs with Mφs without preforming animal study. The target diseases and experimental models are represented in Table 2 in detail.

**Table 2:** Study characteristics and outcomes.

**Author**	**Journal**	**Year**	**Cell source**	**Target disease**	**Study type**	**Cells management**	**Bioengineering method for MSCs paracrine mediators**	**Measurement instrument**
Chang J	Bone Res	2015	C57BL/6 mice	Bone fracture	*In-vitro*	BMSC CM and Mφs cell contract co-culture	BMSCs intrinsic bio-activation: the supernatants from BMSCs cultures were collected and stored at −80 °C until used as conditioned medium.	Scratch assay, BMSCs migration assay, IL-6 ELISA assay, cell growth assay, Gene expression by RT-PCR, Western blot
Cosenza et al., 2017	Sci Rep	2017	C57BL/6 mice	Osteoarthritis	*In-vitro* and *in-vivo*	*In-vitro:* BMSC Exos and Mφs *In-vivo* (arthritis model): IA injections of BM-MSCs, MPs or Exos.	BMSCs intrinsic bio-activation: BMSC-CM was centrifuged at 300 g for 10 min to eliminate cells and 2,500 g for 25 min to remove debris and apoptotic bodies. For MP isolation, CM was centrifuged at 18,000 g for 1 h in polyallomer tubes; the pellet was then suspended in PBS and submitted to a second round of centrifugation. For Exos, supernatant from MP fraction was filtered on 0.22 μm porous membrane and centrifuged at 100,000 g for 2 h.	Flow cytometry analysis, Bone parameter analyses, Confocal laser scanning microscopy, Histological analysis
Lo Sicco	Stem Cells Transl Med	2017	Human-ADSCs; C57BL/6 mice–Mφs	Muscle damage	*In-vitro* and *in-vivo*	*In-vitro:* ADSC EVs and Mφs *In-vivo* (muscle injury model): IL injection of ADSCs EVs	ADSCs extrinsic modification: ADSC EVs were isolated from normoxic- and hypoxic-conditioned media by differential centrifugation at 300 g for 10 min, 2,000 g for 20 min, 10,000 g for 30 min at 4°C to eliminate cells and debris	Protein quantification and immunoblot analysis, Flow cytometry analysis, qRT-PCR, Immunofluorescence analysis, Histology and morphometric analysis
Hyvärinen K	Front Immunol	2018	Human	/	*In-vitro*	BMSCs or BMSC-EVs and Mφs co-culture	BMSCs intrinsic bio-activation: BMSCs media were collected and centrifuged at 2,000 g for 10 min to remove cell debris. The supernatant was ultracentrifuged with Optima™ MAX-XP Ultracentrifuge (Beckman Coulter) at 100,000 g 1.5 h +4°C with MLA-50 rotor (k-factor = 92, Beckman Coulter), and the pelleted EVs were combined. For the second EV collection, the cell starvation was continued in 200 ml α-MEM at 37°C, 5% CO_2_ for 2 days followed by replication of EV centrifugation steps.	Flow cytometry analysis, cytokine (IL-10, IL-23, IL-22) and LMs measurements
Zhang S	Biomaterials	2018	Human embryonic stem cell derived MSCs	Osteoarthritis	*In-vivo*	Sprague-Dawley rat osteochondral defect model: IA injection of MSCs-Exos	BMSCs intrinsic bio-activation: MSCs were grown in a chemically defined medium for 3 days and exosomes were purified from the CM.	Histology and immunohistochemistry, Multiplex cytokine assay (IL-1β, IL-6, TNF-β)
Chamberlain CS	Stem Cells	2019	Human	Tendon injury	*In-vitro* and *in-vivo*	*In-vitro*: BMSC-EVs and Mφs; *In-vivo* (Foxn1nu mouse model of Achilles tendon injury): IL injection of BMSCs, CD14+ Mφs or EEMs	BMSCs intrinsic bio-activation: BMSCs CM was centrifuged using a Beckman Coulter Allegra X-15R centrifuge at 2,000 g at 4°C for 20 min. Clarified supernatant CM was then centrifuged in a Beckman Coulter Optima L-80 XP Ultracentrifuge at 100,000 g at 4°C for 2 h with a swinging bucket SW 28 rotor to pellet EVs.	Flow cytometry analysis, IHC/Immunofluorescence/Histology; Fractal analysis; Mechanical testing
Pacienza N	Mol Ther Methods Clin Dev	2019	Human–BMSCs; RAW 264.7–Mφs cell	/	*In-vitro* and *in-vivo*	*In-vitro*: LPS in combination with Exos and Mφs; *In-vivo* (endotoxemia mouse model): IV injection of Exos	BMSCs intrinsic bio-activation: BMSCs CM was applied directly at room temperature to a column containing the anion exchange resin (Q Sepharose Fast Flow, GE Healthcare, Chicago, IL, USA) that had been equilibrated with 50 mM NaCl in 50 mM phosphate buffer (pH 7.5). The column resin was washed with 100 mM NaCl in 50 mM phosphate buffer (pH 7.5) and then eluted with 500 mM NaCl in 50 mM phosphate buffer (pH 7.5).	qRT-PCR, Quantitation of TNF-a, IL-1β, and IL-6 by ELISA
Shi Z	J Transl Med	2019	Sprague-Dawley rats–BMSCs	Tendon injury	*In-vivo*	Sprague-Dawley rat patellar tendon defect model: injection of fibrin containing EVs	BMSCs CM was centrifuged sequentially at 300 g for 10 min followed by 2,000 g for 10 min to remove cellular debris. The supernatants were then ultracentrifuged at 100,000 g for 2 h to obtain a pellet containing the EVs, which was resuspended in 200 μL of PBS. EVs-enriched fraction was centrifuged at 1,500 g, 30 min with 100-kDa molecular weight cut off (MWCO) hollow fiber membrane (Millipore, Billerica, MA, USA). Then, EVs were passed through a 0.22-μm filter.	Histology and immunohistochemistry, Gene expression, Histological analysis
Li Y	J Cell Biochem	2019	C57BL/6 mice	Bone fracture	*In-vitro* and *in-vivo*	*In-vitro*: ADSCs and Mφs cell contract co-culture;*In-vivo* (a cylindrical bone defect model): IL injection of ADSCs	/	Immunohistochemistry, Western-blot analysis, RT-PCR, Enzyme-linked immunosorbent assay, micro-CT
Shen H	J Orthop Res	2020	Scleraxis-GFP tendon reporter mice or NF-κB-GFP-luciferase transgenic reporter mice–ADSCs Wild type FVB/NJ (FVB) –Mφs	Tendon injury	*In-vitro* and *in-vivo*	*In-vitro:* Exos and Mφs co-culture; *In-vivo* (NGL mouse model of Achilles tendon injury and repair): collagen sheet loaded with EVs from naÃ^_^ve ASCs or IFNγ-primed ASCs	ADSCs intrinsic bio-activation: ADSCs were cultured in an EV collection medium (2% EV-free FBS in α-MEM) for 48 h. CM from ASC culture (150 ml from ~2.5 E+07 cells per isolation) with or without IFNγ pre-treatment was collected and centrifuged at 500 g for 10 min and 10,000 g for 30 min at 4°C to remove large vesicles. After passing through a 0.22 μm filter, the medium was further centrifuged at 100,000 g for 90 min at 4°C.	Mφs assays, NF-κB-Luciferase Imaging *in-vivo*, RT-PCR, Histology

*Both the methodology employed and the results obtained by each article are represented in this table. BMSCs, bone marrow stem cells; ADSCs, adipose tissue derived stem cells; Mφs, macrophages; Exos, exosomes; MPs, microparticles; EVs, extracellular vesicles; CM, conditioned medium; IA, intra-articularly; IL, intralesional; IV, intravenous; LMs, lipid mediators; EEMs, exosome-educated macrophages; LPS, lipopolysaccharide*.

### Cell Sources and Cell Management

Five of 10 studies used human source cells (Lo Sicco et al., 2017; Hyvarinen et al., 2018; Zhang et al., 2018; Chamberlain et al., 2019; Pacienza et al., 2019). Human adipose-derived mesenchymal stem cells (ADSCs) were used for *in vitro* experiments in Lo Sicco's research; the EVs derived from ADSCs were cocultured with mouse Mφs and intralesionally injected into the mouse muscle injury model (Lo Sicco et al., 2017). In Hyvarinen et al. (2018) study, they used human BMSCs or BMSC-derived EVs to coculture with human Mφs. Zhang et al. (2018) intra-articularly injected exosomes from human embryonic stem cell–derived MSCs into rat with osteochondral defect. Chamberlain et al. (2019) also used human BMSCs and Mφs for the *in vitro* experiment. BMSCs, CD14^+^ Mφs and exosome-educated Mφs were intralesionally injected in the mice with Achilles tendon injury. Pacienza et al. (2019) used human BMSCs and RAW 264.7 for the *in vitro* experiment. Exosomes derived from BMSCs were intravenously injected into the endotoxemia mouse model. The *in vitro* experiment cell sources were all C57BL/6 mice in Chang et al. (2015), Cosenza et al. (2017) and Li Y. et al. (2019) studies. Fibrin containing Sprague–Dawley rat BMSC-derived EVs was intralesionally injected into the patellar tendon defect rat in Shi et al. (2019) study. Shen et al. (2020) used an *in vitro* model whose cell source was isolated from specific transgenic mice. The details of cell management are summarized in Table 2.

### Boosting Approaches for MSC-Paracrine Mediators

Lo Sicco et al. (2017) isolated ADSC-derived EVs from normoxic and hypoxic culture condition. Li Y. et al. (2019) used a Transwell coculture system of ADSCs and Mφs without extra management of MSCs paracrine mediators. The other eight studies collected and purified EVs or other paracrine mediators from MSC-derived CM by differential centrifugation approaches (Chang et al., 2015; Cosenza et al., 2017; Lo Sicco et al., 2017; Hyvarinen et al., 2018; Zhang et al., 2018; Chamberlain et al., 2019; Pacienza et al., 2019; Shi et al., 2019; Shen et al., 2020). The detailed methods are described in Table 2.

### Measurement Instruments

For classifications on the results, the measurement instruments, including immunohistochemistry, reverse transcriptase–polymerase chain reaction analysis, Western blot analysis, enzyme-linked immunosorbent assay, flow cytometry analysis, multiplex cytokine assay, confocal laser scanning, micro–computed tomography, and mechanical testing, were used. Also, fractal analysis (Chamberlain et al., 2019) and bone parameter analysis (Cosenza et al., 2017) were applied. Detailed results are listed in Table 2.

### Experimental Variables and Controls

For two bone fracture–related studies, Li Y. et al. (2019) locally injected a total of 1 × 10^6^ ADSCs at the injury site of their cylindrical bone defect model; the therapeutic efficacy of ADSCs was compared to the non-injected group. In the *in vitro* experiment, the bone marrow–derived Mφs were cocultured with 1 × 10^5^ ADSCs in the upper chamber of Transwell under with high-glucose Dulbecco modified eagle medium containing 10% fetal bovine serum (FBS). Chang et al. (2015) planted 1 × 10^5^ bone marrow–derived Mφs into six-well plates and cultured 24 h. Then, 1 × 10^5^ BMSCs or apoptotic BMSCs (exposed to UV light treatment for 30 min) were placed on the Mφs in α-minimum essential medium containing 10% FBS. For each time point, Mφs cultured alone, apoptotic BMSCs cultured alone, and BMSCs cultured alone served as control groups.

For the OA-related studies, Cosenza et al. (2017) applied the *in vitro* OA-like chondrocytes model to investigate the effect of microparticles, BMSC-derived exosomes, BMSC-derived CM, and BMSCs on Mφs in a Transwell system. For their *in vivo* experiment, BMSCs, microparticles, and exosomes were intra-articularly injected into the arthritis model. Those OA mice without treatment served as a control group. In Zhang et al. (2018) study, exosomes and phosphate-buffered saline (PBS) (used as control) were intra-articularly injected into the rat osteochondral defect model after surgery, respectively.

For three tendon injury–related studies, two of them (Chamberlain et al., 2019; Shen et al., 2020) reported the coculture results of EVs and Mφs, and the another one study reported *in vivo* experiment (Shi et al., 2019). Chamberlain et al. (2019) reported that exosome-educated Mφs could be generated by MSC-derived EVs, and Mφs treated with PBS served as control groups in their *in vitro* experiment. To compare the therapeutic potential on tendon healing, the authors intralesionally injected 1 × 10^6^ human BMSCs, 1 × 10^6^ CD14^+^ Mφs, 1 × 10^6^ exosome-educated Mφs, or Hanks balanced saline solution (used as the injured control) to the surgical sites; the contralateral intact Achilles tendon without any treatment was used as a control (Chamberlain et al., 2019). In Shen et al. (2020) study, EVs produced by IFN-γ-primed and non-primed mouse ADSC were used to treat with Mφs to evaluate the impact of EVs on the Mφs inflammatory response. And EV collection medium and EV-free CM were used as controls in the *in vitro* experiment. In Shen et al. (2020) *in vivo* experiment, collagen sheet loaded with EVs was applied around the repair sites, and the collagen sheet only was set as a control group. Shi et al. (2019) placed fibrin glue containing BMSC-EVs in the window defect of patellar tendon. The fibrin glue alone and non-surgical rats were designed as control groups.

In Lo Sicco et al. (2017) study, ADSC-derived EVs and PBS were intramuscularly injected into an injured TA muscle as experimental and control groups, respectively.

No target disease was mentioned in Hyvarinen et al. (2018) and Pacienza et al. (2019) studies. Mφs cocultured with MSCs and MSC-EVs served as experimental variables in Hyvarinen et al. (2018) study, and Mφs served as the control group (Hyvarinen et al., 2018). In Pacienza et al. (2019) study, LPS-stimulated Mφs treated with BMSC-derived exosomes served as the experimental group, and the three control groups were Mφs treated with complete medium alone, LPS, and LPS plus dexamethasone. In Pacienza et al. (2019) *in vivo* experiment, LPS alone or in combination with exosomes (~5 × 10^9^ vesicles) was injected into mice through tail vein, respectively. Control group was injected with saline (Pacienza et al., 2019). Detailed results are listed in Table 3.

**Table 3:** Evaluations and results list of selected studies in which the therapeutic potential of the administration of MSCs for the treatment of musculoskeletal diseases.

**Target disease**	**References**	**Variables**	**Controls**	**Laboratory effects**	**Proposed mechanisms**	**Conclusions**	**Research implications**
Bone fracture	Chang et al., 2015	Seeding BM Mφs first. Then placing primary or apoptotic BMSCs. Additional validation: IL-6 KO mice.	Mφs cultured alone, apoptotic BMSCs cultured alone, and BMSCs cultured alone.	↑ BMSCs migration ↑ BMSCs proliferation	↑ IL-6 proteins and mRNA ! IL-6/gp130/STAT3 pathway	BMSCs are the main contributing cells of juxtacrine IL-6 production. Juxtacrine cultures accelerated BMSCs migration and numbers.	Increase the understanding of Mφs in bone formation.
	Li Y. et al., 2019	ADSCs and BM Mφs co-culture system; ADSCs locally injection.	Untreated control mice	↑ femoral bone formation ↑ femoral bone volume	↑ osteoblasts ↑ CD206+ cells ↑ CD68+ cells ↓ iNOS+ cells ↑ CD11b+ F4/80+ cells ↑ IL-1rα proteins ↓ TNF-α proteins and mRNA ↓ iNOS proteins and mRNA ↑ Arg-1 proteins and mRNA ↑ MR proteins and mRNA ↑ Runx-2 and osterix and OPG and ALP genes ↓ RANKL genes	ADSCs and Mφs can synergistically contribute to bone repair through mutual regulation of their differentiation and cytokine secretion.	The interactions between ADSCs and BM Mφs could be a promising therapeutic strategy in the rehabilitation of bone damage.
Osteoarthritis	Cosenza et al., 2017	Mediums of OA like chondrocytes model were replaced by medium containing MPs, Exos, BMSCs-CM or BMSCs (transwell); BMSCs, MPs or Exos intra-articularly injection.	OA control mice	Restore the anabolic equilibrium ↓ apoptotic chondrocytes ↓ macrophage activation ↑ bone volume, cartilage degradation (surface/volume ratio) and thickness	↓ MMP-13, ADAMTS5, iNOS genes ↑ ACAN, COL2B, COL1 genes ↓ F4/80+ cells ↓ CD86, MHCII or CD40 markers ↓ TNF-α proteins ↑ IL-10 proteins	Exos were more efficient than MPs and BMSCs in chondroprotective and anti-inflammatory function.	MPs and Exos help to promote re-establish chondrocyte homeostatic state.
	Zhang et al., 2018	Intra-articular injection of Exos.	Intra-articular injection of PBS	↑ neotissue formation and ECM deposition of s-GAG ↑ Wakitani score ↑ surface regularity ↑ hyaline cartilage formation ↑ percentage areal deposition of type II collagen ↓ percentage areal deposition of type I collagen ↑ chondrocyte migration, proliferation and matrix synthesis ↑ metabolic activity	↑ chondrocytic cells ↓ PCNA+ cells ↑ CD163+ cells ↓ CD86+ cells ↓ IL-1β and TNF-α ↑ Survivin, Bcl-2, FGF-2 mRNA ! AKT and ERK pathways	Exos mediate cartilage repair by mounting a coordinated, multi-faceted response of enhancing proliferation, migration and matrix synthesis, attenuating apoptosis and modulating immune reactivity.	Exso could be provided as a cell-free MSC therapeutic.
Muscle damage	Lo Sicco et al., 2017	EVs–Mφs co-culture; EVs were intramuscularly administered into muscle.	PBS were intramuscularly administered into muscle	↑ internalization of EVs in Mφs ↑ Mφs proliferation ↓ M1/M2 ↓ mononucleated myoblasts ↑ fibers containing nuclei ≥2	↑ Ly6C, CD11b, CD40, CD86 (post-treat 24h) ↑ CD206, CD51, CD36 (post-treat 72h) ↓ CD11b, CD86 (post-treat 72h) ↓ IL-6/IL-10 ↑ Arg-1, Ym-1markers ↓ Nos-2 markers ↑ CD206+ cells ↓ Ly6C+ cells ↑ MCP-1 ↑ Pax-7, MyoD genes (activated satellite cells); eMyhc gene (regenerating fibers)	EVs co-cultured with responding BM-derived Mφs, shifting the balance toward a M2 phenotype.	Establish an alternative cell-free approach of EVs for the induction of regenerative processes.
Tendon injury	Chamberlain et al., 2019	EVs–Mφs co-culture; BMSCs, CD14+ Mφs or EEMs locally injection.	Mφs controls were treated with PBS; Contralateral controls	↑ EVs educated Mφs ↑ ultimate stress and Young's modulus ↓ M1/M2 ratio ↓ type I collagen ↓ type I/type III collagen ratio	↑ CD206 mean fluorescence intensity and cells ↑ PD-L1 mean fluorescence intensity and cells ↑ M2 Mφs ↓ M1 Mφs ↑ endothelial cells	EVs-educated Mφs treatments improve mechanical properties for tendon function as shown by reduce endogenous M1/M2 ratio indicating less inflammation.	EEMs treatment provides a novel strategy in musculoskeletal injuries.
	Shen et al., 2020	EVs–Mφs co-culture; Collagen sheet loaded with EVs was applied around the repair site.	EVs collection medium and EV-free conditioned medium controls; Collagen sheet only control. IL-1 (EVs from IFNγ-primed ASCs)	↑ NF-κB activity ↑/ ↓ Matrix gene expression ↓ gap-rupture rate	↓ IL-1 gene (only EVs from IFNγ-primed ASCs) ↑ IFNγ gene (only EVs from IFNγ-primed ASCs) ↑ MMP-1, Sox-9 genes ↑ Col-1α1, Col-2α1 and Col-3α1 genes (only EVs from IFNγ-primed ASCs) ↑ collagen staining (only EVs from IFNγ-primed ASCs)	EVs from ASCs can shift the Mφs phenotypic response to tendon injury from a default M1 to a M2 phenotype.	EVs could be a new cell-free therapy, for tendon repair with the potential for improved therapeutic efficacy and safety.
	Shi et al., 2019	Fibrin glue containing EVs was placed in the window defect	Fibrin glue alone and untreated controls	↑ Fiber alignment score ↑ anti-inflammatory response ↑ tendon matrix formation ↑ tenogenesis ↓ tendon cell apoptosis ↑ tendon cell proliferation	↑ CD163 marker ↑ IL-4, IL-10 mRNA and + cells ↓ IFNγ, IL-1β, IL-6, CCR7 + cells ↑ SCX, TNMD, Col-1α1, Col-3α1 genes ↑ CD146+ cells ↓ cleaved caspase 3 signals	EVs derived from BMSCs can help to improve the quality of tendon healing by promoting an anti-inflammatory environment.	These findings provide a basis for the potential clinical use of BMSC-EVs in tendon repair.
None	Hyvarinen et al., 2018	MSCs–Mφs coculture; MSC-EVs–Mφs coculture	Mφs only control	↓ FRI of CD163 ↓ cytokine levels of Mreg-CM ↑ LMs level and pathway markers ↑ phagocytic ability	↓ CD163+ cells ↓ IL-10, IL-22, IL-23, TNF-α proteins ↑ arachidonic acid-derived PGE2, 15-HETE, docosahexaenoic acid-derived 17-HDHA ↑ CD206 Mregs and receptors	Both MSCs and MSC-EVs decrease IL-23 and IL-22 while increasing PGE2 production.	MSC-EVs may potentiate tolerance-promoting proresolving phenotype of human Mregs.
	Pacienza et al., 2019	LPS in combination with Exos–Mφs coculture; Tail vein injection of LPS	FBS medium alone control, containing LPS control, and LPS plus dexamethasone control; Tail vein injection of saline control	↑ anti-inflammatory activity ↑ predictive efficacy	↓ IL-6, IL-1β levels ↓ iNOS mRNA	Exos could suppress LPS-induced inflammation.	*In-vitro* Mφs assay predicts the *in-vivo* anti-inflammatory potential of Exos

*Both the methodology employed and the results obtained by each article are represented in this table. Apoptotic BMSCs (exposed to UV light treatment for 30 min); !, activation; KO, knockout; TNF-α, tumor necrosis factor-α; RANKL, receptor activator of nuclear factor κB ligand; iNOS, inducible nitric oxide synthase; MR, mannose receptor; Arg-1, Antibodies against arginase 1; Runx-2, runt-related transcription factor 2; OPG, osteoprotegerin; ALP, alkaline phosphatase; Mregs, regulatory macrophages; MPs, microparticles; Exos, exosomes; EVs, extracellular vesicles; EEMs, exosome-educated macrophages; OA, osteoarthritis; ECM, extracellular matrix; FRI, fluorescence intensity; LMs, lipid mediators; LPS, lipopolysaccharide*.

### Laboratory Effects and Proposed Mechanisms

The bone fracture–related studies showed that femoral bone formation and bone volume all increased after ADSC injection. The increase in osteoblasts after ADSC injection was accompanied by increases in CD206^+^, CD68^+^, CD11b^+^, and F4/80^+^ cells, which represented a typical M2 surface phenotype. Meanwhile, the transcript and protein expression of Arg-1, M2 marker genes, were also up-regulated (Pacienza et al., 2019). Two OA-related studies reported that bone volume, cartilage thickness, hyaline cartilage formation, migration and proliferation of chondrocytes, and matrix synthesis were increased by BMSC-derived exosomes (Cosenza et al., 2017; Zhang et al., 2018). The therapeutic effect of anti-inflammation on OA was in line with a decrease in M1 Mφs (F4/80^+^, CD86^+^, MHCII^+^, CD40^+^), IL-1β, and tumor necrosis factor α (TNF-α), as well as increases in chondrocytes, M2 Mφs (CD163^+^), and IL-10 (Cosenza et al., 2017; Zhang et al., 2018). Moreover, BMSC-derived exosomes increased type II collagen deposition, and decreased type I collagen in cartilage was reported by Zhang et al. (2018) Such protective effects on chondrocytes might be attributed to the increase in Survivin, Bcl-2, and FGF-2 by BMSC-derived exosomes administration.

Lo Sicco et al. (2017) reported that ADSC-derived EVs were internalized in Mφs at the muscle injury site, which could promote myofiber regeneration. Interestingly, a dynamic change of Mφs subpopulation was observed after transplantation of ADSC-derived EVs. ADSC-derived EVs increased M1 Mφs (Ly6C^+^, CD11b^+^, CD40^+^, CD86^+^) at 24 h post-treatment and increased M2 Mφs (CD206^+^, CD51^+^, CD36^+^) at 72 h post-treatment.

Extended results could be concluded from three tendon injury–related studies (Chamberlain et al., 2019; Shi et al., 2019; Shen et al., 2020). The shift of Mφs from M1 to M2 showed consistent variation to the increase in ultimate stress, Young's modulus (Chamberlain et al., 2019), NF-κB activity (Shen et al., 2020), fiber alignment score, tendon matrix formation, tenogenesis, and tendon cell proliferation (Shi et al., 2019) and the decrease in type I/type III collagen ratio (Chamberlain et al., 2019), gap-rupture rate (Shen et al., 2020), and tendon cell apoptosis (Shi et al., 2019) by BMSC-derived or ADSC-derived exosome administration.

## Discussion

Nowadays, it has been emphatically contended that all newly presented clinical interventions should start with and end with a systematic review, or even meta-analysis (Clarke et al., 2007). Although the therapeutic effects of MSCs via paracrine factors have been demonstrated in many pre-clinical investigations, the substantial clinical evidence in beneficial effects of MSCs in musculoskeletal diseases is not adequately investigated (Regenberg et al., 2009; Lukomska et al., 2019). This systematic review aims to analyze the impact of MSC and MSC-derived EVs on Mφs in the treatment of musculoskeletal diseases. According to the outcomes of the included studies, MSCs could promote musculoskeletal tissue repair or healing via their paracrine regulation on Mφs.

### Interactions of Predominant Mφs With MSC-EVs

Mφs are predominant myeloid cells that chronologically accumulate in musculoskeletal tissue at the onset of injury-induced inflammation and exhibit regulatory activity at all stages of the healing process (Tidball, 2011; Varol et al., 2015). Therefore, Mφs are potent triggers for tissue healing processes, including cell recruitment, proliferation, and remodeling (Artlett, 2013).

**Figure 3:**
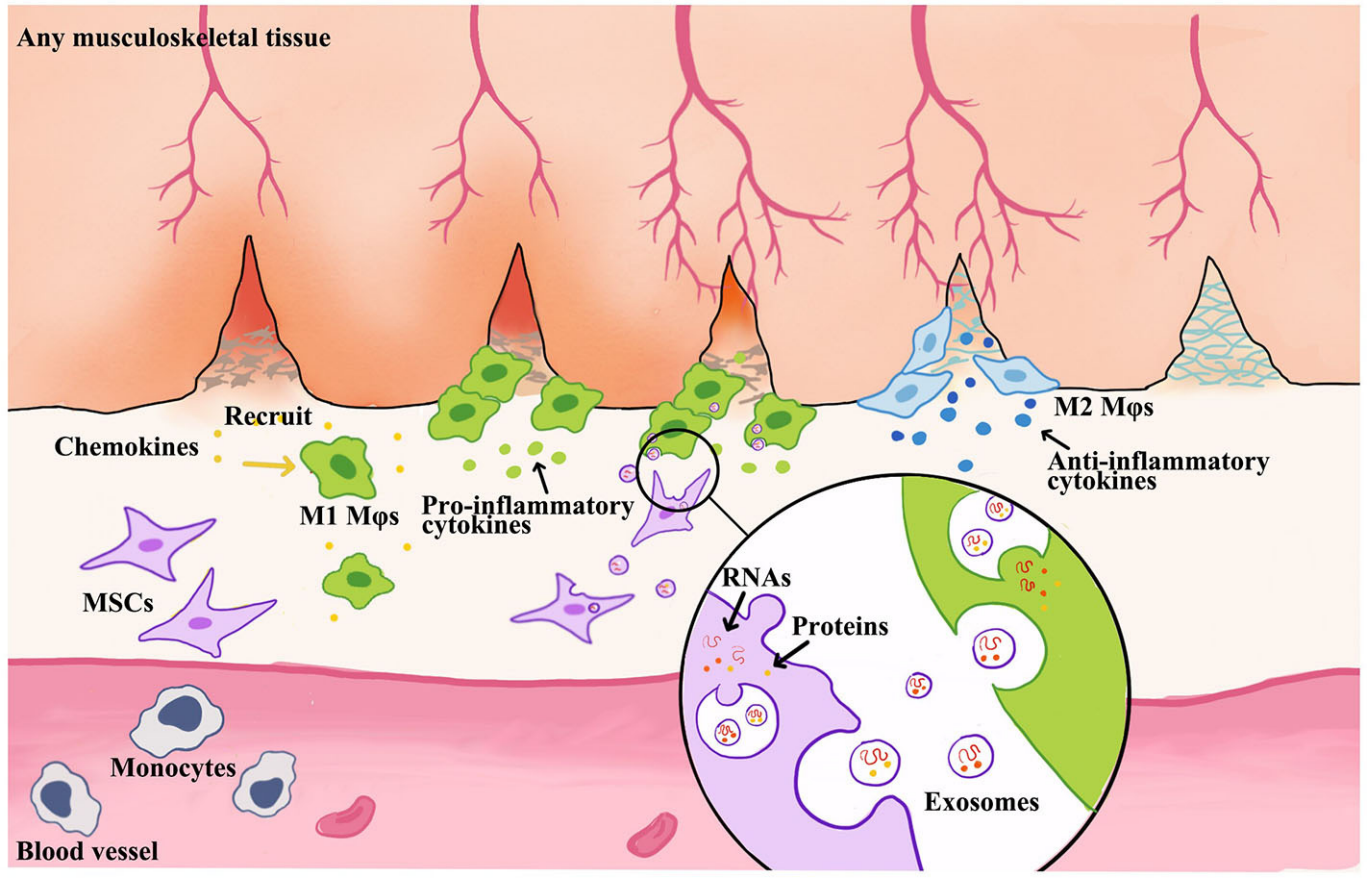
Schematic illustration of MSC-derived exosome-guided macrophage reprogramming. MSC-derived exosomes can induce a conversion of M1 to M2 Mφs and accelerate musculoskeletal tissue healing. Mφs could be activated by inflammatory chemokines and then to produce proinflammatory factors. This creates a feedback loop whereby proinflammatory cytokines produced by Mφs stimulate MSC to produce immune modulators, such as exosomes or EVs. Therefore, the formation of exosomes begins with membrane invagination in the form of endosomes, leading to the development of the early endosomes. Upon maturation, the endosomes become multivesicular endosomes, which release their contents in the form of exosomes.

Growing evidence has demonstrated that the phenotypic switch of Mφs is critical in MSC-mediated tissue regeneration, which is presented in Figure 3. Moreover, M1 Mφ-released proinflammatory cytokines enhanced the migratory capacity of MSCs that facilitates the accessibility of exogenous MSCs toward the injured site. Then, the attracted MSCs would regulate Mφ phenotypes into M2 via paracrine effect to facilitate the tissue remodeling at a later stage (Le Blanc and Mougiakakos, 2012; Maxson et al., 2012; Shi et al., 2012; Bernardo and Fibbe, 2013; Ma et al., 2014). Interestingly, the secretomes of MSCs, such as EVs or exosomes, were also altered by M1 Mφ that boosts the therapeutic effect of MSCs, so-called primed MSCs (Aktas et al., 2017; Saldana et al., 2019).

The cross-talk between Mφs and MSC-derived EVs regulates a shift in Mφs subtypes from M1 into M2 (Galli et al., 2011). An increasing number of musculoskeletal tissue injury models showed that Mφs phenotypic alteration mediated MSC-based therapy (Lo Sicco et al., 2017; Chu et al., 2019). M1 Mφs promote recruitment of inflammatory immune cells and release extracellular matrix (ECM) degrading proteins to allow quick migration to inflamed sites. As the Mφs shift to M2 subtypes, the release of proinflammatory cytokines is inhibited, angiogenesis is stimulated, and fibroblasts are activated to produce and restore more ECM. Also, Mφs induced MSCs into a motile phenotype with increased secretion of IL-6 and IL-10, which benefit MSCs to migrate to injury site (Anton et al., 2012; Wolfe et al., 2016). Typically, MSC-EVs impact the maturation of Mφs by decreasing the expression levels of IL-12 and TNF-α and increasing IL-6 and IL-10 in Mφs (Kim and Hematti, 2009; Cosenza et al., 2017; Lo Sicco et al., 2017; Hyvarinen et al., 2018; Shi et al., 2019). PGE_2_ and TNF-α inducible gene 6 (TSG-6) embedded in MSC-EVs can promote M2 polarization in inflammatory microenvironment (Nemeth et al., 2009; Maggini et al., 2010; Melief et al., 2013b). EVs, which were released from proinflammatory cytokines–activated MSCs, could enhance anti-inflammatory properties to suppress MHC class II and CD86 signaling in LPS-stimulated Mφs (Melief et al., 2013a,b; Cho et al., 2014). Lo Sicco et al. (2017) and Chamberlain et al. (2019) reported the MSC-EVs change the M1-to-M2 Mφs ratio. And Lo Sicco et al. (2017) demonstrated that MSC-EVs could be efficiently internalized by responding cells, inducing an increase in their proliferation rate, and shifting the balance toward an alternatively anti-inflammatory M2 phenotype. M2 Mφs phenotype could be commonly associated with the secretion of the anti-inflammatory cytokine IL-10 and scavenger receptors CD206 and CD163 (Sica and Mantovani, 2012; Mantovani et al., 2013).

In this review, we found consistent results suggesting that an M2 phenotype could be induced from M1 Mφs upon coculture with MSCs, MSC-EVs, and CM. The enrichment of M1 Mφs appears at early phases (1–3 days) during bone remodeling (Chang et al., 2015; Li Y. et al., 2019), muscle regeneration (Lo Sicco et al., 2017), and tendon healing (Chamberlain et al., 2019; Harvey et al., 2019; Shi et al., 2019; Shen et al., 2020) and is later replaced by M2 Mφs (4–7 days). As Mφ polarization and tissue repair by MSC-EVs are highly associated, MSC-EVs–mediated Mφs phenotypic transformation must play a significant role during tissue healing. However, the molecular action of MSC-EVs in such Mφ polarization and tissue regeneration needs further investigation (Chen et al., 2008; Rodero and Khosrotehrani, 2010).

Although studies have provided evidence of immunosuppressive effects of MSCs in clinical trials for graft-vs.-host disease and Crohn disease (Godoy et al., 2019), the transplanted MSCs have not been proven in the persistence after injection and the contribution in tissue regeneration (Pittenger, 2009; Parekkadan and Milwid, 2010; Caplan and Correa, 2011). It is likely that the main immunosuppressive effects of MSCs resulted from paracrine regulation through secreted mediators, including EVs (Raposo and Stoorvogel, 2013). The immune modulation effect of MSC has been generalized as the inhibition of both innate and adaptive immunity and also derives inflammatory, autoimmune, and infectious disease pathology (Le Blanc and Mougiakakos, 2012; English, 2013). However, increasing analysis in animal models of inflammation demonstrated that MSC-EVs suppressed immune response through the transfer of RNAs and protein (Cantaluppi et al., 2012; Arslan et al., 2013). The inflammatory signals are essential to initiate and maintain the MSC- or EV-mediated tissue repair process; afterward, MSC-derived EVs could manipulate the niche by switching Mφ phenotype to facilitate tissue repair.

### Functional MSC-EVs Cargo and Boosting Approaches

Recently, the use of MSC-based therapies has emerged. The therapeutic benefits of MSC transplantation have been attributed into two types, EVs and soluble factors. Soluble components include a wide variety of secreted chemokines, growth factors, and hormones with immunomodulatory activity. For example, PGE_2_ and TGF-β are vital mediators of anti-inflammatory in therapeutic therapy of MSCs (Yoo et al., 2013). And lots of anti-inflammatory proteins also represent tissue protection, such as TSG-6, which has been reported with healing ability by reducing the influx of neutrophils to the tissue injury site (Oh et al., 2010). The studies regarding MSC-derived EVs have grown exponentially since it has been recognized that EVs containing mRNA, miRNA, and protein could exchange intracellular information and act as sophisticated mediators of recipient cell behavior, particularly in immunomodulation (Wolfers et al., 2001; Valadi et al., 2007; Skog et al., 2008). MSC-derived EVs contain not only more than 200 mRNA and 60 miRNA, but also more than 800 proteins (Bruno et al., 2009; Chen et al., 2010; Lai et al., 2012). These EV-derived proteins have been reported with an integral role in activating anti-inflammatory responses and regulating the cascade of tissue healing process in various injury models (Lee et al., 2012; Zhang et al., 2018; Li et al., 2019).

It has been demonstrated that genetically modified MSCs possess great prosurvival, proangiogenic, and anti-inflammatory properties not only by the altering release of soluble proteins, but also EVs (Huang et al., 2013; Wang et al., 2016; Lou et al., 2017; Ma et al., 2017; Qu et al., 2017; Tao et al., 2017). MSC-derived EVs are engineered at the cellular level under natural conditions, and it further highlighted the unique advantage of EV-based nanoplatforms for cargo delivery (Luan et al., 2017). For clinical applications, some advantages of MSC-derived EVs include easier injection, reduced Mφs phagocytosis and vascular occlusion (EL Andaloussi et al., 2013), innate biocompatibility, high physicochemical stability, high penetrability, and long-distance communication (Clayton et al., 2003; Ridder et al., 2014; Zomer et al., 2015).

Many strategies have been applied to modify EVs, including cell modification and direct EV modification (Armstrong and Perriman, 2016). Because EVs are secreted from cells, they can intrinsically express some lipids or cell adhesion molecules and ligands that naturally target certain types of recipient cells (Luan et al., 2017). Undoubtedly, it was inevitable that genetic engineering has been used to modify EVs. Overexpression of mRNA or miRNA in cells could be assembled into EVs, which could be fused to target cells to introduce or inhibit gene expression (Kosaka et al., 2010; Akao et al., 2011; Ridder et al., 2014; Zomer et al., 2015). For a significant amount of time, researchers have explored non-native biomaterials to cells to augment therapeutic function (Armstrong et al., 2015; Correia Carreira et al., 2016). Therefore, biomaterials delivered to the membrane could also naturally be incorporated into budding EVs, while internalized material may be packaged into exosomes for secretion (Armstrong et al., 2017). Taking advantage of these methods allows cellular processes and cell engineering techniques to be specifically adapted to EV functionalization.

Because only a small fraction of material could be packaged into EVs, the efficiency issue results in a low-yielding ending. And besides the widely explored cancer cells, the yield of exosomes derived from MSCs is one of the major factors that limits the expansion of cell-free therapeutic productions (Phan et al., 2018). Functional EVs could be derived from native ECM (Huleihel et al., 2016; Du et al., 2017), and three-dimensional environment for cell attachment and growth has been particularly attracted because it can mimic the ECM structure and function (Phan et al., 2018). Such structure has been reported with the effects of influencing EV secretion. For example, Tao et al. reported that Avitene Ultrafoam collagen hemostat caused the BMSCs to release 2-fold of exosomes compared to the plastic surface culture based on protein assay (Tao et al., 2017). Besides, a bioactive artificial ECM that was modified by adding molecules has been used to imitate native ECM mimicking structures and to improve yield of MSC exosomes by presenting specific functional ligands (Hao et al., 2017).

Moreover, an essential and urgent solution can provide an approach to improve the yield without sacrificing the functionality or with enhancing the efficacy simultaneously. Therefore, researchers tried to purify EVs, which could ensure that all modified sites or encapsulated species could be localized at the vesicle (Armstrong et al., 2017). As non-living entities, EVs have a major advantage over cells when they received membrane surface modification. It has been reported that excessive pressures, temperature, chemical induction, or hypoxia environment exposure could cause membrane disruption, vesicle aggregation, and surface protein denaturation (Smyth et al., 2014; Armstrong et al., 2017; Lo Sicco et al., 2017). Also, multivalent electrostatic interactions, receptor–ligand binding, and hydrophobic insertion have been commonly applied as methods of biological membrane modifications (Nakase and Futaki, 2015; Correia Carreira et al., 2016; Lee et al., 2016). In addition, electroporation, which is an alternative approach to EV active loading strategies, has been reported to transiently permeabilize the EV membrane to enhance the absorptivity of small molecules (Tian et al., 2014; Fuhrmann et al., 2015). Taken together, the engineered EVs will open up exciting opportunities in EV-based therapies by boosting therapeutic capability, which is beyond their native functions.

## Strengths and Limitations

This systematic review poses several advantages compared to other attempts to summarize the experimental results of MSC-derived paracrine mediators for musculoskeletal diseases. First, the up-to-date literature search has been yielded by two widely used databases: PubMed and EMBASE. The search strategies included MeSH terms and other related terms. Therefore, we could identify a number of eligible studies, which might remain relatively unnoticed. Second, the quality of included studies and risk of bias, including publication bias, were assessed. Third, study heterogeneity had been explored to point out potential explanatory variables. Fourth, to standardize the spontaneous recovery in the control groups, we analyzed the variables and controls specifically. And studies without scientific controls have been excluded during full-text screening procedure.

There were several limitations to this study. The first limitation is the small number of included studies. The second limitation is that we could not standardize the effectiveness of Mφs depletion among different studies. Moreover, the depletion is not permanent and, once subsided, could result in a reactive increase in Mφs numbers with unknown consequences. Third, these studies utilized different cell sources and delivery methods. Different types of animals were selected for *in vivo* studies. Fourth, the assessment methods also widely varied among studies; therefore, it was impossible to perform a quantitative analysis or a meta-analysis with the included studies.

## Conclusion

This review demonstrated that MSC and MSC-EVs are authentic biomaterials to treat musculoskeletal problems. The broad therapeutic effect of MSC and MSC-EVs attribute to the management of Mφ polarization, at least in part. A further understanding in the molecular mechanism of how MSCs regulate Mφ polarization will facilitate the development of bioengineering approach to boost the therapeutic capacity of MSCs and their clinical application. However, more pre-clinical studies are needed to understand how EVs and their subcomponent play a role in musculoskeletal tissue healing process.

## Data Availability Statement

All datasets generated for this study are included in the article/Supplementary Material.

## Author Contributions

HX: conception and design, manuscript writing, and data analysis and interpretation. C-WL: conception and design, financial support, and manuscript writing. Y-FW: manuscript writing. L-YS, Y-HW, ZW, and XZ: data analysis and interpretation. PY and OL: administrative support and final approval of manuscript. SH: revisions to scientific content of manuscripts. All authors contributed to the article and approved the submitted version.

## Conflict of Interest

The authors declare that the research was conducted in the absence of any commercial or financial relationships that could be construed as a potential conflict of interest.
