# Supplementary material for: The Role of Paracrine Regulation of Mesenchymal Stem Cells in the Crosstalk With Macrophages in Musculoskeletal Diseases: A Systematic Review
Source: Front Bioeng Biotechnol. 2020 Nov 26;8:587052. doi: 10.3389/fbioe.2020.587052 (PMC7726268; doi:10.3389/fbioe.2020.587052)
Supplement: Supplementary file 1 [file Data_Sheet_1.DOCX]

***Appendix 1***

***Mesh term:***

Mesenchymal Stem Cells

Exosomes

Extracellular Vesicles

Condition Medium

Macrophages

***Free words:***

- Stem Cell, Mesenchymal
- Stem Cells, Mesenchymal
- Mesenchymal Stem Cell
- Bone Marrow Mesenchymal Stem Cells
- Bone Marrow Stromal Cells
- Bone Marrow Stromal Cell
- Bone Marrow Stromal Cells, Multipotent
- Multipotent Bone Marrow Stromal Cells
- Adipose-Derived Mesenchymal Stem Cells
- Adipose Derived Mesenchymal Stem Cells
- Mesenchymal Stem Cells, Adipose-Derived
- Mesenchymal Stem Cells, Adipose Derived
- Adipose-Derived Mesenchymal Stromal Cells
- Adipose Derived Mesenchymal Stromal Cells
- Adipose Tissue-Derived Mesenchymal Stem Cells
- Adipose Tissue Derived Mesenchymal Stem Cells
- Adipose Tissue-Derived Mesenchymal Stromal Cells
- Adipose Tissue Derived Mesenchymal Stromal Cells
- Mesenchymal Stromal Cells
- Mesenchymal Stromal Cell
- Stromal Cell, Mesenchymal
- Stromal Cells, Mesenchymal
- Multipotent Mesenchymal Stromal Cells
- Mesenchymal Stromal Cells, Multipotent
- Mesenchymal Progenitor Cell
- Mesenchymal Progenitor Cells
- Progenitor Cell, Mesenchymal
- Progenitor Cells, Mesenchymal
- Wharton Jelly Cells
- Wharton's Jelly Cells
- Wharton's Jelly Cell
- Whartons Jelly Cells
- Bone Marrow Stromal Stem Cells
- Endosomes
- Secretory Vesicles
- Cell-Derived Microparticles
- Exosome Multienzyme Ribonuclease Complex
- Extracellular Vesicle
- Vesicle, Extracellular
- Vesicles, Extracellular
- Exovesicles
- Exovesicle
- Apoptotic Bodies
- Apoptotic Body
- Bodies, Apoptotic
- Body, Apoptotic
- Conditioned Medium
- Medium, Conditioned
- Culture Medium, Conditioned
- Conditioned Culture Media
- Conditioned Media
- Media, Conditioned
- Conditioned Culture Medium
- Bone Marrow-Derived Macrophages
- Bone Marrow Derived Macrophages
- Bone Marrow-Derived Macrophage
- Macrophage, Bone Marrow-Derived
- Macrophages, Bone Marrow-Derived
- Monocyte-Derived Macrophages
- Monocyte Derived Macrophages
- Macrophage
- Macrophages, Monocyte-Derived
- Macrophage, Monocyte-Derived
- Macrophages, Monocyte Derived
- Monocyte-Derived Macrophage

Appendix 2

Recent queries in PubMed
Search, Query, Items found, Time

| [#11](https://www.ncbi.nlm.nih.gov/pubmed/advanced) | [Add](https://www.ncbi.nlm.nih.gov/pubmed/advanced) | Search **("****Mesenchymal Stem Cells"[Mesh]) OR (((((((((((((((((((((((((((((((((((Mesenchymal Stem Cells[Title/Abstract]) OR Mesenchymal Stem Cell[Title/Abstract]) OR Stem Cell, Mesenchymal[Title/Abstract]) OR Stem Cells, Mesenchymal[Title/Abstract]) OR Mesenchymal Stem Cell[Title/Abstract]) OR Wharton Jelly Cells[Title/Abstract]) OR Wharton's Jelly Cells[Title/Abstract]) OR Wharton's Jelly Cell[Title/Abstract]) OR Whartons Jelly Cells[Title/Abstract]) OR Mesenchymal Stromal Cells[Title/Abstract]) OR Mesenchymal Stromal Cell[Title/Abstract]) OR Stromal Cell, Mesenchymal[Title/Abstract]) OR Stromal Cells, Mesenchymal[Title/Abstract]) OR Adipose-Derived Mesenchymal Stem Cells[Title/Abstract]) OR Adipose Derived Mesenchymal Stem Cells[Title/Abstract]) OR Adipose Tissue-Derived Mesenchymal Stem Cells[Title/Abstract]) OR Adipose Tissue Derived Mesenchymal Stem Cells[Title/Abstract]) OR Adipose-Derived Mesenchymal Stromal Cells[Title/Abstract]) OR Adipose Derived Mesenchymal Stromal Cells[Title/Abstract]) OR Adipose Tissue-Derived Mesenchymal Stromal Cells[Title/Abstract]) OR Adipose Tissue Derived Mesenchymal Stromal Cells[Title/Abstract]) OR Mesenchymal Stem Cells, Adipose-Derived[Title/Abstract]) OR Mesenchymal Stem Cells, Adipose Derived[Title/Abstract]) OR Bone Marrow Stromal Cells, Multipotent[Title/Abstract]) OR Multipotent Bone Marrow Stromal Cells[Title/Abstract]) OR Bone Marrow Stromal Stem Cells[Title/Abstract]) OR Multipotent Mesenchymal Stromal Cells[Title/Abstract]) OR Mesenchymal Stromal Cells, Multipotent[Title/Abstract]) OR Mesenchymal Progenitor Cell[Title/Abstract]) OR Mesenchymal Progenitor Cells[Title/Abstract]) OR Progenitor Cell, Mesenchymal[Title/Abstract]) OR Progenitor Cells, Mesenchymal[Title/Abstract]) OR Bone Marrow Mesenchymal Stem Cells[Title/Abstract]) OR Bone Marrow Stromal Cells[Title/Abstract]) OR Bone Marrow Stromal Cell[Title/Abstract])** | 69525 | 07:12:32 |
| --- | --- | --- | --- | --- |
| [#10](https://www.ncbi.nlm.nih.gov/pubmed/advanced) | [Add](https://www.ncbi.nlm.nih.gov/pubmed/advanced) | Search **((((((((((((((((((((((((((((((((((Mesenchymal Stem Cells[Title/Abstract]) OR Mesenchymal Stem Cell[Title/Abstract]) OR Stem Cell, Mesenchymal[Title/Abstract]) OR Stem Cells, Mesenchymal[Title/Abstract]) OR Mesenchymal Stem Cell[Title/Abstract]) OR Wharton Jelly Cells[Title/Abstract]) OR Wharton's Jelly Cells[Title/Abstract]) OR Wharton's Jelly Cell[Title/Abstract]) OR Whartons Jelly Cells[Title/Abstract]) OR Mesenchymal Stromal Cells[Title/Abstract]) OR Mesenchymal Stromal Cell[Title/Abstract]) OR Stromal Cell, Mesenchymal[Title/Abstract]) OR Stromal Cells, Mesenchymal[Title/Abstract]) OR Adipose-Derived Mesenchymal Stem Cells[Title/Abstract]) OR Adipose Derived Mesenchymal Stem Cells[Title/Abstract]) OR Adipose Tissue-Derived Mesenchymal Stem Cells[Title/Abstract]) OR Adipose Tissue Derived Mesenchymal Stem Cells[Title/Abstract]) OR Adipose-Derived Mesenchymal Stromal Cells[Title/Abstract]) OR Adipose Derived Mesenchymal Stromal Cells[Title/Abstract]) OR Adipose Tissue-Derived Mesenchymal Stromal Cells[Title/Abstract]) OR Adipose Tissue Derived Mesenchymal Stromal Cells[Title/Abstract]) OR Mesenchymal Stem Cells, Adipose-Derived[Title/Abstract]) OR Mesenchymal Stem Cells, Adipose Derived[Title/Abstract]) OR Bone Marrow Stromal Cells, Multipotent[Title/Abstract]) OR Multipotent Bone Marrow Stromal Cells[Title/Abstract]) OR Bone Marrow Stromal Stem Cells[Title/Abstract]) OR Multipotent Mesenchymal Stromal Cells[Title/Abstract]) OR Mesenchymal Stromal Cells, Multipotent[Title/Abstract]) OR Mesenchymal Progenitor Cell[Title/Abstract]) OR Mesenchymal Progenitor Cells[Title/Abstract]) OR Progenitor Cell, Mesenchymal[Title/Abstract]) OR Progenitor Cells, Mesenchymal[Title/Abstract]) OR Bone Marrow Mesenchymal Stem Cells[Title/Abstract]) OR Bone Marrow Stromal Cells[Title/Abstract]) OR Bone Marrow Stromal Cell[Title/Abstract]** | 66643 | 07:11:32 |
| [#9](https://www.ncbi.nlm.nih.gov/pubmed/advanced) | [Add](https://www.ncbi.nlm.nih.gov/pubmed/advanced) | Search **"Mesenchymal Stem Cells"[Mesh]** | 34792 | 06:57:59 |

| [#43](https://www.ncbi.nlm.nih.gov/pubmed/advanced) | [Add](https://www.ncbi.nlm.nih.gov/pubmed/advanced) | Search **("Exosomes"[Mesh]) OR ((((Endosomes[Title/Abstract]) OR Secretory Vesicles[Title/Abstract]) OR Cell-Derived Microparticles[Title/Abstract]) OR Exosome Multienzyme Ribonuclease Complex[Title/Abstract])** | 21544 | 11:59:46 |
| --- | --- | --- | --- | --- |
| [#42](https://www.ncbi.nlm.nih.gov/pubmed/advanced) | [Add](https://www.ncbi.nlm.nih.gov/pubmed/advanced) | Search **(((Endosomes[Title/Abstract]) OR Secretory Vesicles[Title/Abstract]) OR Cell-Derived Microparticles[Title/Abstract]) OR Exosome Multienzyme Ribonuclease Complex[Title/Abstract]** | 16121 | 11:59:28 |
| [#41](https://www.ncbi.nlm.nih.gov/pubmed/advanced) | [Add](https://www.ncbi.nlm.nih.gov/pubmed/advanced) | Search **"Exosomes"[Mesh]** | 5572 | 11:59:00 |

| [#5](https://www.ncbi.nlm.nih.gov/pubmed/advanced) | [Add](https://www.ncbi.nlm.nih.gov/pubmed/advanced) | Search **(****"****Extracellular Vesicles"[Mesh]) OR (((((((((Extracellular Vesicle[Title/Abstract]) OR Vesicle, Extracellular[Title/Abstract]) OR Vesicles, Extracellular[Title/Abstract]) OR Exovesicles[Title/Abstract]) OR Exovesicle[Title/Abstract]) OR Apoptotic Bodies[Title/Abstract]) OR Apoptotic Body[Title/Abstract]) OR Bodies, Apoptotic[Title/Abstract]) OR Body, Apoptotic[Title/Abstract])** | 27244 | 21:23:39 |
| --- | --- | --- | --- | --- |
| [#4](https://www.ncbi.nlm.nih.gov/pubmed/advanced) | [Add](https://www.ncbi.nlm.nih.gov/pubmed/advanced) | Search **((((((((Extracellular Vesicle[Title/Abstract]) OR Vesicle, Extracellular[Title/Abstract]) OR Vesicles, Extracellular[Title/Abstract]) OR Exovesicles[Title/Abstract]) OR Exovesicle[Title/Abstract]) OR Apoptotic Bodies[Title/Abstract]) OR** **Apoptotic Body[Title/Abstract]) OR Bodies, Apoptotic[Title/Abstract]) OR Body, Apoptotic[Title/Abstract]** | 20618 | 21:23:30 |
| [#3](https://www.ncbi.nlm.nih.gov/pubmed/advanced) | [Add](https://www.ncbi.nlm.nih.gov/pubmed/advanced) | Search **"Extracellular Vesicles"[Mesh]** | 9955 | 21:22:13 |

| [#12](https://www.ncbi.nlm.nih.gov/pubmed/advanced) | [Add](https://www.ncbi.nlm.nih.gov/pubmed/advanced) | Search **("Culture Media, Conditioned"[Mesh]) OR (((((((Conditioned Medium[Title/Abstract]) OR Medium, Conditioned[Title/Abstract]) OR Culture Medium, Conditioned[Title/Abstract]) OR Conditioned Culture Media[Title/Abstract]) OR Conditioned Media[Title/Abstract]) OR Media, Conditioned[Title/Abstract]) OR Conditioned Culture Medium[Title/Abstract])** | 35531 | 05:27:03 |
| --- | --- | --- | --- | --- |
| [#10](https://www.ncbi.nlm.nih.gov/pubmed/advanced) | [Add](https://www.ncbi.nlm.nih.gov/pubmed/advanced) | Search **((((((Conditioned Medium[Title/Abstract]) OR Medium, Conditioned[Title/Abstract]) OR Culture Medium, Conditioned[Title/Abstract]) OR Conditioned Culture Media[Title/Abstract]) OR Conditioned Media[Title/Abstract]) OR Media, Conditioned[Title/Abstract]) OR Conditioned Culture Medium[Title/Abstract]** | 29077 | 05:26:13 |
| [#11](https://www.ncbi.nlm.nih.gov/pubmed/advanced) | [Add](https://www.ncbi.nlm.nih.gov/pubmed/advanced) | Search **"****Culture Media, Conditioned"[Mesh]** | 14405 | 05:26:50 |

| [#13](https://www.ncbi.nlm.nih.gov/pubmed/advanced) | [Add](https://www.ncbi.nlm.nih.gov/pubmed/advanced) | Search **(((("Exosomes"[Mesh]) OR ((((Endosomes[Title/Abstract]) OR Secretory Vesicles[Title/Abstract]) OR Cell-Derived Microparticles[Title/Abstract]) OR Exosome Multienzyme Ribonuclease Complex[Title/Abstract]))) OR (("Extracellular Vesicles"[Mesh]) OR (((((((((Extracellular Vesicle[Title/Abstract]) OR Vesicle, Extracellular[Title/Abstract]) OR Vesicles, Extracellular[Title/Abstract]) OR Exovesicles[Title/Abstract]) OR Exovesicle[Title/Abstract]) OR Apoptotic Bodies[Title/Abstract]) OR Apoptotic Body[Title/Abstract]) OR Bodies, Apoptotic[Title/Abstract]) OR Body, Apoptotic[Title/Abstract]))) OR (("Culture Media, Conditioned"[Mesh]) OR (((((((Conditioned Medium[Title/Abstract]) OR Medium, Conditioned[Title/Abstract]) OR Culture Medium, Conditioned[Title/Abstract]) OR Conditioned Culture Media[Title/Abstract]) OR Conditioned Media[Title/Abstract]) OR Media, Conditioned[Title/Abstract]) OR Conditioned Culture Medium[Title/Abstract]))** | [77316](https://www.ncbi.nlm.nih.gov/pubmed/?cmd=HistorySearch&querykey=13) | 05:29:24 |
| --- | --- | --- | --- | --- |

| [#14](https://www.ncbi.nlm.nih.gov/pubmed/advanced) | [Add](https://www.ncbi.nlm.nih.gov/pubmed/advanced) | Search **((("Mesenchymal Stem Cells"[Mesh]) OR (((((((((((((((((((((((((((((((((((Mesenchymal Stem Cells[Title/Abstract]) OR Mesenchymal Stem Cell[Title/Abstract]) OR Stem Cell, Mesenchymal[Title/Abstract]) OR Stem Cells, Mesenchymal[Title/Abstract]) OR Mesenchymal Stem Cell[Title/Abstract]) OR Wharton Jelly Cells[Title/Abstract]) OR Wharton's Jelly Cells[Title/Abstract]) OR Wharton's Jelly Cell[Title/Abstract]) OR Whartons Jelly Cells[Title/Abstract]) OR Mesenchymal Stromal Cells[Title/Abstract]) OR Mesenchymal Stromal Cell[Title/Abstract]) OR Stromal Cell, Mesenchymal[Title/Abstract]) OR Stromal Cells, Mesenchymal[Title/Abstract]) OR Adipose-Derived Mesenchymal Stem Cells[Title/Abstract]) OR Adipose Derived Mesenchymal Stem Cells[Title/Abstract]) OR Adipose Tissue-Derived Mesenchymal Stem Cells[Title/Abstract]) OR Adipose Tissue Derived Mesenchymal Stem Cells[Title/Abstract]) OR Adipose-Derived Mesenchymal Stromal Cells[Title/Abstract]) OR Adipose Derived Mesenchymal Stromal Cells[Title/Abstract]) OR Adipose Tissue-Derived Mesenchymal Stromal Cells[Title/Abstract]) OR Adipose Tissue Derived Mesenchymal Stromal Cells[Title/Abstract]) OR Mesenchymal Stem Cells, Adipose-Derived[Title/Abstract]) OR Mesenchymal Stem Cells, Adipose Derived[Title/Abstract]) OR Bone Marrow Stromal Cells, Multipotent[Title/Abstract]) OR Multipotent Bone Marrow Stromal Cells[Title/Abstract]) OR Bone Marrow Stromal Stem Cells[Title/Abstract]) OR Multipotent Mesenchymal Stromal Cells[Title/Abstract]) OR Mesenchymal Stromal Cells, Multipotent[Title/Abstract]) OR Mesenchymal Progenitor Cell[Title/Abstract]) OR Mesenchymal Progenitor Cells[Title/Abstract]) OR Progenitor Cell, Mesenchymal[Title/Abstract]) OR Progenitor Cells, Mesenchymal[Title/Abstract]) OR Bone Marrow Mesenchymal Stem Cells[Title/Abstract]) OR Bone Marrow Stromal Cells[Title/Abstract]) OR Bone Marrow Stromal Cell[Title/Abstract]))) AND ((((("Exosomes"[Mesh]) OR ((((Endosomes[Title/Abstract]) OR Secretory Vesicles[Title/Abstract]) OR Cell-Derived Microparticles[Title/Abstract]) OR Exosome Multienzyme Ribonuclease Complex[Title/Abstract]))) OR (("Extracellular Vesicles"[Mesh]) OR (((((((((Extracellular Vesicle[Title/Abstract]) OR Vesicle, Extracellular[Title/Abstract]) OR Vesicles, Extracellular[Title/Abstract]) OR Exovesicles[Title/Abstract]) OR Exovesicle[Title/Abstract]) OR Apoptotic Bodies[Title/Abstract]) OR Apoptotic Body[Title/Abstract]) OR Bodies, Apoptotic[Title/Abstract]) OR Body, Apoptotic[Title/Abstract]))) OR (("Culture Media, Conditioned"[Mesh]) OR (((((((Conditioned Medium[Title/Abstract]) OR Medium, Conditioned[Title/Abstract]) OR Culture Medium, Conditioned[Title/Abstract]) OR Conditioned Culture Media[Title/Abstract]) OR Conditioned Media[Title/Abstract]) OR Media, Conditioned[Title/Abstract]) OR Conditioned Culture Medium[Title/Abstract])))** | 4131 | 05:30:52 |
| --- | --- | --- | --- | --- |

| [#17](https://www.ncbi.nlm.nih.gov/pubmed/advanced) | [Add](https://www.ncbi.nlm.nih.gov/pubmed/advanced) | Search **("Macrophages"[Mesh]) OR ((((((((((((Bone Marrow-Derived Macrophages[Title/Abstract]) OR Bone Marrow Derived Macrophages[Title/Abstract]) OR Bone Marrow-Derived Macrophage[Title/Abstract]) OR Macrophage, Bone Marrow-Derived[Title/Abstract]) OR Macrophages, Bone Marrow-Derived[Title/Abstract]) OR Monocyte-Derived Macrophages[Title/Abstract]) OR Monocyte Derived Macrophages[Title/Abstract]) OR Macrophage[Title/Abstract]) OR Macrophages, Monocyte-Derived[Title/Abstract]) OR Macrophage, Monocyte-Derived[Title/Abstract]) OR Macrophages, Monocyte Derived[Title/Abstract]) OR Monocyte-Derived Macrophage[Title/Abstract])** | 244560 | 07:17:46 |
| --- | --- | --- | --- | --- |
| [#16](https://www.ncbi.nlm.nih.gov/pubmed/advanced) | [Add](https://www.ncbi.nlm.nih.gov/pubmed/advanced) | Search **(((((((((((Bone Marrow-Derived Macrophages[Title/Abstract]) OR Bone Marrow Derived Macrophages[Title/Abstract]) OR Bone Marrow-Derived Macrophage[Title/Abstract]) OR Macrophage, Bone Marrow-Derived[Title/Abstract]) OR Macrophages, Bone Marrow-Derived[Title/Abstract]) OR Monocyte-Derived Macrophages[Title/Abstract]) OR Monocyte Derived Macrophages[Title/Abstract]) OR Macrophage[Title/Abstract]) OR Macrophages, Monocyte-Derived[Title/Abstract]) OR Macrophage, Monocyte-Derived[Title/Abstract]) OR Macrophages, Monocyte Derived[Title/Abstract]) OR Monocyte-Derived Macrophage[Title/Abstract]** | 135641 | 07:17:35 |
| [#15](https://www.ncbi.nlm.nih.gov/pubmed/advanced) | [Add](https://www.ncbi.nlm.nih.gov/pubmed/advanced) | Search **"Macrophages"[Mesh]** | 178000 | 07:14:07 |

| [#15](https://www.ncbi.nlm.nih.gov/pubmed/advanced) | [Add](https://www.ncbi.nlm.nih.gov/pubmed/advanced) | Search **((((("Mesenchymal Stem Cells"[Mesh]) OR (((((((((((((((((((((((((((((((((((Mesenchymal Stem Cells[Title/Abstract]) OR Mesenchymal Stem Cell[Title/Abstract]) OR Stem Cell, Mesenchymal[Title/Abstract]) OR Stem Cells, Mesenchymal[Title/Abstract]) OR Mesenchymal Stem Cell[Title/Abstract]) OR Wharton Jelly Cells[Title/Abstract]) OR Wharton's Jelly Cells[Title/Abstract]) OR Wharton's Jelly Cell[Title/Abstract]) OR Whartons Jelly Cells[Title/Abstract]) OR Mesenchymal Stromal Cells[Title/Abstract]) OR Mesenchymal Stromal Cell[Title/Abstract]) OR Stromal Cell, Mesenchymal[Title/Abstract]) OR Stromal Cells, Mesenchymal[Title/Abstract]) OR Adipose-Derived Mesenchymal Stem Cells[Title/Abstract]) OR Adipose Derived Mesenchymal Stem Cells[Title/Abstract]) OR Adipose Tissue-Derived Mesenchymal Stem Cells[Title/Abstract]) OR Adipose Tissue Derived Mesenchymal Stem Cells[Title/Abstract]) OR Adipose-Derived Mesenchymal Stromal Cells[Title/Abstract]) OR Adipose Derived Mesenchymal Stromal Cells[Title/Abstract]) OR Adipose Tissue-Derived Mesenchymal Stromal Cells[Title/Abstract]) OR Adipose Tissue Derived Mesenchymal Stromal Cells[Title/Abstract]) OR Mesenchymal Stem Cells, Adipose-Derived[Title/Abstract]) OR Mesenchymal Stem Cells, Adipose Derived[Title/Abstract]) OR Bone Marrow Stromal Cells, Multipotent[Title/Abstract]) OR Multipotent Bone Marrow Stromal Cells[Title/Abstract]) OR Bone Marrow Stromal Stem Cells[Title/Abstract]) OR Multipotent Mesenchymal Stromal Cells[Title/Abstract]) OR Mesenchymal Stromal Cells, Multipotent[Title/Abstract]) OR Mesenchymal Progenitor Cell[Title/Abstract]) OR Mesenchymal Progenitor Cells[Title/Abstract]) OR Progenitor Cell, Mesenchymal[Title/Abstract]) OR Progenitor Cells, Mesenchymal[Title/Abstract]) OR Bone Marrow Mesenchymal Stem Cells[Title/Abstract]) OR Bone Marrow Stromal Cells[Title/Abstract]) OR Bone Marrow Stromal Cell[Title/Abstract]))) AND ((((("Exosomes"[Mesh]) OR ((((Endosomes[Title/Abstract]) OR Secretory Vesicles[Title/Abstract]) OR Cell-Derived Microparticles[Title/Abstract]) OR Exosome Multienzyme Ribonuclease Complex[Title/Abstract]))) OR (("Extracellular Vesicles"[Mesh]) OR (((((((((Extracellular Vesicle[Title/Abstract]) OR Vesicle, Extracellular[Title/Abstract]) OR Vesicles, Extracellular[Title/Abstract]) OR Exovesicles[Title/Abstract]) OR Exovesicle[Title/Abstract]) OR Apoptotic Bodies[Title/Abstract]) OR Apoptotic Body[Title/Abstract]) OR Bodies, Apoptotic[Title/Abstract]) OR Body, Apoptotic[Title/Abstract]))) OR (("Culture Media, Conditioned"[Mesh]) OR (((((((Conditioned Medium[Title/Abstract]) OR Medium, Conditioned[Title/Abstract]) OR Culture Medium, Conditioned[Title/Abstract]) OR Conditioned Culture Media[Title/Abstract]) OR Conditioned Media[Title/Abstract]) OR Media, Conditioned[Title/Abstract]) OR Conditioned Culture Medium[Title/Abstract]))))) AND (("Macrophages"[Mesh]) OR ((((((((((((Bone Marrow-Derived Macrophages[Title/Abstract]) OR Bone Marrow Derived Macrophages[Title/Abstract]) OR Bone Marrow-Derived Macrophage[Title/Abstract]) OR Macrophage, Bone Marrow-Derived[Title/Abstract]) OR Macrophages, Bone Marrow-Derived[Title/Abstract]) OR Monocyte-Derived Macrophages[Title/Abstract]) OR Monocyte Derived Macrophages[Title/Abstract]) OR Macrophage[Title/Abstract]) OR Macrophages, Monocyte-Derived[Title/Abstract]) OR Macrophage, Monocyte-Derived[Title/Abstract]) OR Macrophages, Monocyte Derived[Title/Abstract]) OR Monocyte-Derived Macrophage[Title/Abstract]))** | 331 | 05:31:59 |
| --- | --- | --- | --- | --- |

Appendix 3

Embase
Session Results
.......................................................

| \| Search history sorted by search number ascending \| \| \| \| \| \| \| \| --- \| --- \| --- \| --- \| --- \| --- \| --- \| \|  \| [# ▲](http://ovidsp.dc1.ovid.com/sp-4.02.1a/ovidweb.cgi?&S=CEFBFPHJKLACKGKCKPCKFHHOANPJAA00&Sort+Sets=descending) \| **Searches** \| **Results** \| **Type** \| **Actions** \| **Annotations** \| \|  \| \| \| \| \| \| \| \|  \| 1 \| Stem Cell, Mesenchymal.mp. \| 33 \| Advanced \| [Display Results](http://ovidsp.dc1.ovid.com/sp-4.02.1a/ovidweb.cgi?&S=CEFBFPHJKLACKGKCKPCKFHHOANPJAA00&SELECT=S.sh%7c&R=1&Process+Action=display)  More \| [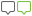](http://ovidsp.dc1.ovid.com/sp-4.02.1a/ovidweb.cgi?&S=CEFBFPHJKLACKGKCKPCKFHHOANPJAA00&R=1&Search+Annotations+Options=SA) \| \|  \| 2 \| Stem Cells, Mesenchymal.mp. \| 270 \| Advanced \| [Display Results](http://ovidsp.dc1.ovid.com/sp-4.02.1a/ovidweb.cgi?&S=CEFBFPHJKLACKGKCKPCKFHHOANPJAA00&SELECT=S.sh%7c&R=2&Process+Action=display)  More \| [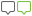](http://ovidsp.dc1.ovid.com/sp-4.02.1a/ovidweb.cgi?&S=CEFBFPHJKLACKGKCKPCKFHHOANPJAA00&R=2&Search+Annotations+Options=SA) \| \|  \| 3 \| Mesenchymal Stem Cell.mp. \| 64264 \| Advanced \| [Display Results](http://ovidsp.dc1.ovid.com/sp-4.02.1a/ovidweb.cgi?&S=CEFBFPHJKLACKGKCKPCKFHHOANPJAA00&SELECT=S.sh%7c&R=3&Process+Action=display)  More \| [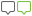](http://ovidsp.dc1.ovid.com/sp-4.02.1a/ovidweb.cgi?&S=CEFBFPHJKLACKGKCKPCKFHHOANPJAA00&R=3&Search+Annotations+Options=SA) \| \|  \| 4 \| Wharton Jelly Cells.mp. \| 5 \| Advanced \| [Display Results](http://ovidsp.dc1.ovid.com/sp-4.02.1a/ovidweb.cgi?&S=CEFBFPHJKLACKGKCKPCKFHHOANPJAA00&SELECT=S.sh%7c&R=4&Process+Action=display)  More \| [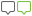](http://ovidsp.dc1.ovid.com/sp-4.02.1a/ovidweb.cgi?&S=CEFBFPHJKLACKGKCKPCKFHHOANPJAA00&R=4&Search+Annotations+Options=SA) \| \|  \| 5 \| Wharton's Jelly Cells.mp. \| 39 \| Advanced \| [Display Results](http://ovidsp.dc1.ovid.com/sp-4.02.1a/ovidweb.cgi?&S=CEFBFPHJKLACKGKCKPCKFHHOANPJAA00&SELECT=S.sh%7c&R=5&Process+Action=display)  More \| [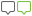](http://ovidsp.dc1.ovid.com/sp-4.02.1a/ovidweb.cgi?&S=CEFBFPHJKLACKGKCKPCKFHHOANPJAA00&R=5&Search+Annotations+Options=SA) \| \|  \| 6 \| Wharton's Jelly Cell.mp. \| 2 \| Advanced \| [Display Results](http://ovidsp.dc1.ovid.com/sp-4.02.1a/ovidweb.cgi?&S=CEFBFPHJKLACKGKCKPCKFHHOANPJAA00&SELECT=S.sh%7c&R=6&Process+Action=display)  More \| [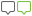](http://ovidsp.dc1.ovid.com/sp-4.02.1a/ovidweb.cgi?&S=CEFBFPHJKLACKGKCKPCKFHHOANPJAA00&R=6&Search+Annotations+Options=SA) \| \|  \| 7 \| Whartons Jelly Cells.mp. \| 39 \| Advanced \| [Display Results](http://ovidsp.dc1.ovid.com/sp-4.02.1a/ovidweb.cgi?&S=CEFBFPHJKLACKGKCKPCKFHHOANPJAA00&SELECT=S.sh%7c&R=7&Process+Action=display)  More \| [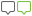](http://ovidsp.dc1.ovid.com/sp-4.02.1a/ovidweb.cgi?&S=CEFBFPHJKLACKGKCKPCKFHHOANPJAA00&R=7&Search+Annotations+Options=SA) \| \|  \| 8 \| Mesenchymal Stromal Cells.mp. \| 9094 \| Advanced \| [Display Results](http://ovidsp.dc1.ovid.com/sp-4.02.1a/ovidweb.cgi?&S=CEFBFPHJKLACKGKCKPCKFHHOANPJAA00&SELECT=S.sh%7c&R=8&Process+Action=display)  More \| [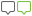](http://ovidsp.dc1.ovid.com/sp-4.02.1a/ovidweb.cgi?&S=CEFBFPHJKLACKGKCKPCKFHHOANPJAA00&R=8&Search+Annotations+Options=SA) \| \|  \| 9 \| Mesenchymal Stromal Cell.mp. \| 2164 \| Advanced \| [Display Results](http://ovidsp.dc1.ovid.com/sp-4.02.1a/ovidweb.cgi?&S=CEFBFPHJKLACKGKCKPCKFHHOANPJAA00&SELECT=S.sh%7c&R=9&Process+Action=display)  More \| [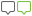](http://ovidsp.dc1.ovid.com/sp-4.02.1a/ovidweb.cgi?&S=CEFBFPHJKLACKGKCKPCKFHHOANPJAA00&R=9&Search+Annotations+Options=SA) \| \|  \| 10 \| Stromal Cell, Mesenchymal.mp. \| 6 \| Advanced \| [Display Results](http://ovidsp.dc1.ovid.com/sp-4.02.1a/ovidweb.cgi?&S=CEFBFPHJKLACKGKCKPCKFHHOANPJAA00&SELECT=S.sh%7c&R=10&Process+Action=display)  More \| [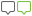](http://ovidsp.dc1.ovid.com/sp-4.02.1a/ovidweb.cgi?&S=CEFBFPHJKLACKGKCKPCKFHHOANPJAA00&R=10&Search+Annotations+Options=SA) \| \|  \| 11 \| Stromal Cells, Mesenchymal.mp. \| 48 \| Advanced \| [Display Results](http://ovidsp.dc1.ovid.com/sp-4.02.1a/ovidweb.cgi?&S=CEFBFPHJKLACKGKCKPCKFHHOANPJAA00&SELECT=S.sh%7c&R=11&Process+Action=display)  More \| [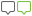](http://ovidsp.dc1.ovid.com/sp-4.02.1a/ovidweb.cgi?&S=CEFBFPHJKLACKGKCKPCKFHHOANPJAA00&R=11&Search+Annotations+Options=SA) \| \|  \| 12 \| Adipose-Derived Mesenchymal Stem Cells.mp. \| 1824 \| Advanced \| [Display Results](http://ovidsp.dc1.ovid.com/sp-4.02.1a/ovidweb.cgi?&S=CEFBFPHJKLACKGKCKPCKFHHOANPJAA00&SELECT=S.sh%7c&R=12&Process+Action=display)  More \| [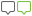](http://ovidsp.dc1.ovid.com/sp-4.02.1a/ovidweb.cgi?&S=CEFBFPHJKLACKGKCKPCKFHHOANPJAA00&R=12&Search+Annotations+Options=SA) \| \|  \| 13 \| Adipose Derived Mesenchymal Stem Cells.mp. \| 1824 \| Advanced \| [Display Results](http://ovidsp.dc1.ovid.com/sp-4.02.1a/ovidweb.cgi?&S=CEFBFPHJKLACKGKCKPCKFHHOANPJAA00&SELECT=S.sh%7c&R=13&Process+Action=display)  More \| [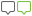](http://ovidsp.dc1.ovid.com/sp-4.02.1a/ovidweb.cgi?&S=CEFBFPHJKLACKGKCKPCKFHHOANPJAA00&R=13&Search+Annotations+Options=SA) \| \|  \| 14 \| Adipose Tissue-Derived Mesenchymal Stem Cells.mp. \| 1002 \| Advanced \| [Display Results](http://ovidsp.dc1.ovid.com/sp-4.02.1a/ovidweb.cgi?&S=CEFBFPHJKLACKGKCKPCKFHHOANPJAA00&SELECT=S.sh%7c&R=14&Process+Action=display)  More \| [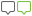](http://ovidsp.dc1.ovid.com/sp-4.02.1a/ovidweb.cgi?&S=CEFBFPHJKLACKGKCKPCKFHHOANPJAA00&R=14&Search+Annotations+Options=SA) \| \|  \| 15 \| Adipose Tissue Derived Mesenchymal Stem Cells.mp. \| 1002 \| Advanced \| [Display Results](http://ovidsp.dc1.ovid.com/sp-4.02.1a/ovidweb.cgi?&S=CEFBFPHJKLACKGKCKPCKFHHOANPJAA00&SELECT=S.sh%7c&R=15&Process+Action=display)  More \| [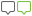](http://ovidsp.dc1.ovid.com/sp-4.02.1a/ovidweb.cgi?&S=CEFBFPHJKLACKGKCKPCKFHHOANPJAA00&R=15&Search+Annotations+Options=SA) \| \|  \| 16 \| Adipose-Derived Mesenchymal Stromal Cells.mp. \| 170 \| Advanced \| [Display Results](http://ovidsp.dc1.ovid.com/sp-4.02.1a/ovidweb.cgi?&S=CEFBFPHJKLACKGKCKPCKFHHOANPJAA00&SELECT=S.sh%7c&R=16&Process+Action=display)  More \| [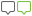](http://ovidsp.dc1.ovid.com/sp-4.02.1a/ovidweb.cgi?&S=CEFBFPHJKLACKGKCKPCKFHHOANPJAA00&R=16&Search+Annotations+Options=SA) \| \|  \| 17 \| Adipose Derived Mesenchymal Stromal Cells.mp. \| 170 \| Advanced \| [Display Results](http://ovidsp.dc1.ovid.com/sp-4.02.1a/ovidweb.cgi?&S=CEFBFPHJKLACKGKCKPCKFHHOANPJAA00&SELECT=S.sh%7c&R=17&Process+Action=display)  More \| [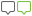](http://ovidsp.dc1.ovid.com/sp-4.02.1a/ovidweb.cgi?&S=CEFBFPHJKLACKGKCKPCKFHHOANPJAA00&R=17&Search+Annotations+Options=SA) \| \|  \| 18 \| Adipose Tissue-Derived Mesenchymal Stromal Cells.mp. \| 123 \| Advanced \| [Display Results](http://ovidsp.dc1.ovid.com/sp-4.02.1a/ovidweb.cgi?&S=CEFBFPHJKLACKGKCKPCKFHHOANPJAA00&SELECT=S.sh%7c&R=18&Process+Action=display)  More \| [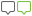](http://ovidsp.dc1.ovid.com/sp-4.02.1a/ovidweb.cgi?&S=CEFBFPHJKLACKGKCKPCKFHHOANPJAA00&R=18&Search+Annotations+Options=SA) \| \|  \| 19 \| Adipose Tissue Derived Mesenchymal Stromal Cells.mp. \| 123 \| Advanced \| [Display Results](http://ovidsp.dc1.ovid.com/sp-4.02.1a/ovidweb.cgi?&S=CEFBFPHJKLACKGKCKPCKFHHOANPJAA00&SELECT=S.sh%7c&R=19&Process+Action=display)  More \| [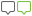](http://ovidsp.dc1.ovid.com/sp-4.02.1a/ovidweb.cgi?&S=CEFBFPHJKLACKGKCKPCKFHHOANPJAA00&R=19&Search+Annotations+Options=SA) \| \|  \| 20 \| Mesenchymal Stem Cells, Adipose-Derived.mp. \| 27 \| Advanced \| [Display Results](http://ovidsp.dc1.ovid.com/sp-4.02.1a/ovidweb.cgi?&S=CEFBFPHJKLACKGKCKPCKFHHOANPJAA00&SELECT=S.sh%7c&R=20&Process+Action=display)  More \| [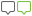](http://ovidsp.dc1.ovid.com/sp-4.02.1a/ovidweb.cgi?&S=CEFBFPHJKLACKGKCKPCKFHHOANPJAA00&R=20&Search+Annotations+Options=SA) \| \|  \| 21 \| Mesenchymal Stem Cells, Adipose Derived.mp. \| 27 \| Advanced \| [Display Results](http://ovidsp.dc1.ovid.com/sp-4.02.1a/ovidweb.cgi?&S=CEFBFPHJKLACKGKCKPCKFHHOANPJAA00&SELECT=S.sh%7c&R=21&Process+Action=display)  More \| [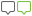](http://ovidsp.dc1.ovid.com/sp-4.02.1a/ovidweb.cgi?&S=CEFBFPHJKLACKGKCKPCKFHHOANPJAA00&R=21&Search+Annotations+Options=SA) \| \|  \| 22 \| Bone Marrow Stromal Cells, Multipotent.mp. \| 1 \| Advanced \| [Display Results](http://ovidsp.dc1.ovid.com/sp-4.02.1a/ovidweb.cgi?&S=CEFBFPHJKLACKGKCKPCKFHHOANPJAA00&SELECT=S.sh%7c&R=22&Process+Action=display)  More \| [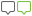](http://ovidsp.dc1.ovid.com/sp-4.02.1a/ovidweb.cgi?&S=CEFBFPHJKLACKGKCKPCKFHHOANPJAA00&R=22&Search+Annotations+Options=SA) \| \|  \| 23 \| Multipotent Bone Marrow Stromal Cells.mp. \| 18 \| Advanced \| [Display Results](http://ovidsp.dc1.ovid.com/sp-4.02.1a/ovidweb.cgi?&S=CEFBFPHJKLACKGKCKPCKFHHOANPJAA00&SELECT=S.sh%7c&R=23&Process+Action=display)  More \| [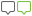](http://ovidsp.dc1.ovid.com/sp-4.02.1a/ovidweb.cgi?&S=CEFBFPHJKLACKGKCKPCKFHHOANPJAA00&R=23&Search+Annotations+Options=SA) \| \|  \| 24 \| Bone Marrow Stromal Stem Cells.mp. \| 302 \| Advanced \| [Display Results](http://ovidsp.dc1.ovid.com/sp-4.02.1a/ovidweb.cgi?&S=CEFBFPHJKLACKGKCKPCKFHHOANPJAA00&SELECT=S.sh%7c&R=24&Process+Action=display)  More \| [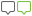](http://ovidsp.dc1.ovid.com/sp-4.02.1a/ovidweb.cgi?&S=CEFBFPHJKLACKGKCKPCKFHHOANPJAA00&R=24&Search+Annotations+Options=SA) \| \|  \| 25 \| Multipotent Mesenchymal Stromal Cells.mp. \| 882 \| Advanced \| [Display Results](http://ovidsp.dc1.ovid.com/sp-4.02.1a/ovidweb.cgi?&S=CEFBFPHJKLACKGKCKPCKFHHOANPJAA00&SELECT=S.sh%7c&R=25&Process+Action=display)  More \| [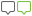](http://ovidsp.dc1.ovid.com/sp-4.02.1a/ovidweb.cgi?&S=CEFBFPHJKLACKGKCKPCKFHHOANPJAA00&R=25&Search+Annotations+Options=SA) \| \|  \| 26 \| Mesenchymal Stromal Cells, Multipotent.mp. \| 3 \| Advanced \| [Display Results](http://ovidsp.dc1.ovid.com/sp-4.02.1a/ovidweb.cgi?&S=CEFBFPHJKLACKGKCKPCKFHHOANPJAA00&SELECT=S.sh%7c&R=26&Process+Action=display)  More \| [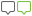](http://ovidsp.dc1.ovid.com/sp-4.02.1a/ovidweb.cgi?&S=CEFBFPHJKLACKGKCKPCKFHHOANPJAA00&R=26&Search+Annotations+Options=SA) \| \|  \| 27 \| Mesenchymal Progenitor Cell.mp. \| 267 \| Advanced \| [Display Results](http://ovidsp.dc1.ovid.com/sp-4.02.1a/ovidweb.cgi?&S=CEFBFPHJKLACKGKCKPCKFHHOANPJAA00&SELECT=S.sh%7c&R=27&Process+Action=display)  More \| [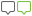](http://ovidsp.dc1.ovid.com/sp-4.02.1a/ovidweb.cgi?&S=CEFBFPHJKLACKGKCKPCKFHHOANPJAA00&R=27&Search+Annotations+Options=SA) \| \|  \| 28 \| Mesenchymal Progenitor Cells.mp. \| 1188 \| Advanced \| [Display Results](http://ovidsp.dc1.ovid.com/sp-4.02.1a/ovidweb.cgi?&S=CEFBFPHJKLACKGKCKPCKFHHOANPJAA00&SELECT=S.sh%7c&R=28&Process+Action=display)  More \| [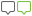](http://ovidsp.dc1.ovid.com/sp-4.02.1a/ovidweb.cgi?&S=CEFBFPHJKLACKGKCKPCKFHHOANPJAA00&R=28&Search+Annotations+Options=SA) \| \|  \| 29 \| Progenitor Cell, Mesenchymal.mp. \| 6 \| Advanced \| [Display Results](http://ovidsp.dc1.ovid.com/sp-4.02.1a/ovidweb.cgi?&S=CEFBFPHJKLACKGKCKPCKFHHOANPJAA00&SELECT=S.sh%7c&R=29&Process+Action=display)  More \| [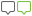](http://ovidsp.dc1.ovid.com/sp-4.02.1a/ovidweb.cgi?&S=CEFBFPHJKLACKGKCKPCKFHHOANPJAA00&R=29&Search+Annotations+Options=SA) \| \|  \| 30 \| Progenitor Cells, Mesenchymal.mp. \| 54 \| Advanced \| [Display Results](http://ovidsp.dc1.ovid.com/sp-4.02.1a/ovidweb.cgi?&S=CEFBFPHJKLACKGKCKPCKFHHOANPJAA00&SELECT=S.sh%7c&R=30&Process+Action=display)  More \| [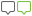](http://ovidsp.dc1.ovid.com/sp-4.02.1a/ovidweb.cgi?&S=CEFBFPHJKLACKGKCKPCKFHHOANPJAA00&R=30&Search+Annotations+Options=SA) \| \|  \| 31 \| Bone Marrow Mesenchymal Stem Cells.mp. \| 7505 \| Advanced \| [Display Results](http://ovidsp.dc1.ovid.com/sp-4.02.1a/ovidweb.cgi?&S=CEFBFPHJKLACKGKCKPCKFHHOANPJAA00&SELECT=S.sh%7c&R=31&Process+Action=display)  More \| [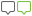](http://ovidsp.dc1.ovid.com/sp-4.02.1a/ovidweb.cgi?&S=CEFBFPHJKLACKGKCKPCKFHHOANPJAA00&R=31&Search+Annotations+Options=SA) \| \|  \| 32 \| Bone Marrow Stromal Cells.mp. \| 7549 \| Advanced \| [Display Results](http://ovidsp.dc1.ovid.com/sp-4.02.1a/ovidweb.cgi?&S=CEFBFPHJKLACKGKCKPCKFHHOANPJAA00&SELECT=S.sh%7c&R=32&Process+Action=display)  More \| [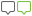](http://ovidsp.dc1.ovid.com/sp-4.02.1a/ovidweb.cgi?&S=CEFBFPHJKLACKGKCKPCKFHHOANPJAA00&R=32&Search+Annotations+Options=SA) \| \|  \| 33 \| Bone Marrow Stromal Cell.mp. \| 2128 \| Advanced \| [Display Results](http://ovidsp.dc1.ovid.com/sp-4.02.1a/ovidweb.cgi?&S=CEFBFPHJKLACKGKCKPCKFHHOANPJAA00&SELECT=S.sh%7c&R=33&Process+Action=display)  More \| [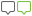](http://ovidsp.dc1.ovid.com/sp-4.02.1a/ovidweb.cgi?&S=CEFBFPHJKLACKGKCKPCKFHHOANPJAA00&R=33&Search+Annotations+Options=SA) \| \|  \| 34 \| 1 or 2 or 3 or 4 or 5 or 6 or 7 or 8 or 9 or 10 or 11 or 12 or 13 or 14 or 15 or 16 or 17 or 18 or 19 or 20 or 21 or 22 or 23 or 24 or 25 or 26 or 27 or 28 or 29 or 30 or 31 or 32 or 33 \| 81005 \| Advanced \| [Display Results](http://ovidsp.dc1.ovid.com/sp-4.02.1a/ovidweb.cgi?&S=CEFBFPHJKLACKGKCKPCKFHHOANPJAA00&SELECT=S.sh%7c&R=34&Process+Action=display)  More \| [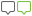](http://ovidsp.dc1.ovid.com/sp-4.02.1a/ovidweb.cgi?&S=CEFBFPHJKLACKGKCKPCKFHHOANPJAA00&R=34&Search+Annotations+Options=SA) \| \|  \| 35 \| Endosomes.mp. \| 15534 \| Advanced \| [Display Results](http://ovidsp.dc1.ovid.com/sp-4.02.1a/ovidweb.cgi?&S=CEFBFPHJKLACKGKCKPCKFHHOANPJAA00&SELECT=S.sh%7c&R=35&Process+Action=display)  More \| [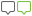](http://ovidsp.dc1.ovid.com/sp-4.02.1a/ovidweb.cgi?&S=CEFBFPHJKLACKGKCKPCKFHHOANPJAA00&R=35&Search+Annotations+Options=SA) \| \|  \| 36 \| Secretory Vesicles.mp. \| 3445 \| Advanced \| [Display Results](http://ovidsp.dc1.ovid.com/sp-4.02.1a/ovidweb.cgi?&S=CEFBFPHJKLACKGKCKPCKFHHOANPJAA00&SELECT=S.sh%7c&R=36&Process+Action=display)  More \| [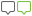](http://ovidsp.dc1.ovid.com/sp-4.02.1a/ovidweb.cgi?&S=CEFBFPHJKLACKGKCKPCKFHHOANPJAA00&R=36&Search+Annotations+Options=SA) \| \|  \| 37 \| Cell-Derived Microparticles.mp. \| 551 \| Advanced \| [Display Results](http://ovidsp.dc1.ovid.com/sp-4.02.1a/ovidweb.cgi?&S=CEFBFPHJKLACKGKCKPCKFHHOANPJAA00&SELECT=S.sh%7c&R=37&Process+Action=display)  More \| [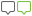](http://ovidsp.dc1.ovid.com/sp-4.02.1a/ovidweb.cgi?&S=CEFBFPHJKLACKGKCKPCKFHHOANPJAA00&R=37&Search+Annotations+Options=SA) \| \|  \| 38 \| Exosome Multienzyme Ribonuclease Complex.mp. \| 72 \| Advanced \| [Display Results](http://ovidsp.dc1.ovid.com/sp-4.02.1a/ovidweb.cgi?&S=CEFBFPHJKLACKGKCKPCKFHHOANPJAA00&SELECT=S.sh%7c&R=38&Process+Action=display)  More \| [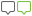](http://ovidsp.dc1.ovid.com/sp-4.02.1a/ovidweb.cgi?&S=CEFBFPHJKLACKGKCKPCKFHHOANPJAA00&R=38&Search+Annotations+Options=SA) \| \|  \| 39 \| 35 or 36 or 37 or 38 \| 19491 \| Advanced \| [Display Results](http://ovidsp.dc1.ovid.com/sp-4.02.1a/ovidweb.cgi?&S=CEFBFPHJKLACKGKCKPCKFHHOANPJAA00&SELECT=S.sh%7c&R=39&Process+Action=display)  More \| [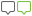](http://ovidsp.dc1.ovid.com/sp-4.02.1a/ovidweb.cgi?&S=CEFBFPHJKLACKGKCKPCKFHHOANPJAA00&R=39&Search+Annotations+Options=SA) \| \|  \| 40 \| Extracellular Vesicle.mp. \| 1985 \| Advanced \| [Display Results](http://ovidsp.dc1.ovid.com/sp-4.02.1a/ovidweb.cgi?&S=CEFBFPHJKLACKGKCKPCKFHHOANPJAA00&SELECT=S.sh%7c&R=40&Process+Action=display)  More \| [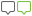](http://ovidsp.dc1.ovid.com/sp-4.02.1a/ovidweb.cgi?&S=CEFBFPHJKLACKGKCKPCKFHHOANPJAA00&R=40&Search+Annotations+Options=SA) \| \|  \| 41 \| Vesicle, Extracellular.mp. \| 3 \| Advanced \| [Display Results](http://ovidsp.dc1.ovid.com/sp-4.02.1a/ovidweb.cgi?&S=CEFBFPHJKLACKGKCKPCKFHHOANPJAA00&SELECT=S.sh%7c&R=41&Process+Action=display)  More \| [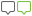](http://ovidsp.dc1.ovid.com/sp-4.02.1a/ovidweb.cgi?&S=CEFBFPHJKLACKGKCKPCKFHHOANPJAA00&R=41&Search+Annotations+Options=SA) \| \|  \| 42 \| Vesicles, Extracellular.mp. \| 31 \| Advanced \| [Display Results](http://ovidsp.dc1.ovid.com/sp-4.02.1a/ovidweb.cgi?&S=CEFBFPHJKLACKGKCKPCKFHHOANPJAA00&SELECT=S.sh%7c&R=42&Process+Action=display)  More \| [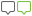](http://ovidsp.dc1.ovid.com/sp-4.02.1a/ovidweb.cgi?&S=CEFBFPHJKLACKGKCKPCKFHHOANPJAA00&R=42&Search+Annotations+Options=SA) \| \|  \| 43 \| Exovesicles.mp. \| 42 \| Advanced \| [Display Results](http://ovidsp.dc1.ovid.com/sp-4.02.1a/ovidweb.cgi?&S=CEFBFPHJKLACKGKCKPCKFHHOANPJAA00&SELECT=S.sh%7c&R=43&Process+Action=display)  More \| [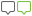](http://ovidsp.dc1.ovid.com/sp-4.02.1a/ovidweb.cgi?&S=CEFBFPHJKLACKGKCKPCKFHHOANPJAA00&R=43&Search+Annotations+Options=SA) \| \|  \| 44 \| Exovesicle.mp. \| 10 \| Advanced \| [Display Results](http://ovidsp.dc1.ovid.com/sp-4.02.1a/ovidweb.cgi?&S=CEFBFPHJKLACKGKCKPCKFHHOANPJAA00&SELECT=S.sh%7c&R=44&Process+Action=display)  More \| [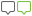](http://ovidsp.dc1.ovid.com/sp-4.02.1a/ovidweb.cgi?&S=CEFBFPHJKLACKGKCKPCKFHHOANPJAA00&R=44&Search+Annotations+Options=SA) \| \|  \| 45 \| Apoptotic Bodies.mp. \| 4269 \| Advanced \| [Display Results](http://ovidsp.dc1.ovid.com/sp-4.02.1a/ovidweb.cgi?&S=CEFBFPHJKLACKGKCKPCKFHHOANPJAA00&SELECT=S.sh%7c&R=45&Process+Action=display)  More \| [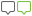](http://ovidsp.dc1.ovid.com/sp-4.02.1a/ovidweb.cgi?&S=CEFBFPHJKLACKGKCKPCKFHHOANPJAA00&R=45&Search+Annotations+Options=SA) \| \|  \| 46 \| Apoptotic Body.mp. \| 782 \| Advanced \| [Display Results](http://ovidsp.dc1.ovid.com/sp-4.02.1a/ovidweb.cgi?&S=CEFBFPHJKLACKGKCKPCKFHHOANPJAA00&SELECT=S.sh%7c&R=46&Process+Action=display)  More \| [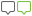](http://ovidsp.dc1.ovid.com/sp-4.02.1a/ovidweb.cgi?&S=CEFBFPHJKLACKGKCKPCKFHHOANPJAA00&R=46&Search+Annotations+Options=SA) \| \|  \| 47 \| Bodies, Apoptotic.mp. \| 36 \| Advanced \| [Display Results](http://ovidsp.dc1.ovid.com/sp-4.02.1a/ovidweb.cgi?&S=CEFBFPHJKLACKGKCKPCKFHHOANPJAA00&SELECT=S.sh%7c&R=47&Process+Action=display)  More \| [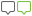](http://ovidsp.dc1.ovid.com/sp-4.02.1a/ovidweb.cgi?&S=CEFBFPHJKLACKGKCKPCKFHHOANPJAA00&R=47&Search+Annotations+Options=SA) \| \|  \| 48 \| Body, Apoptotic.mp. \| 5 \| Advanced \| [Display Results](http://ovidsp.dc1.ovid.com/sp-4.02.1a/ovidweb.cgi?&S=CEFBFPHJKLACKGKCKPCKFHHOANPJAA00&SELECT=S.sh%7c&R=48&Process+Action=display)  More \| [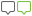](http://ovidsp.dc1.ovid.com/sp-4.02.1a/ovidweb.cgi?&S=CEFBFPHJKLACKGKCKPCKFHHOANPJAA00&R=48&Search+Annotations+Options=SA) \| \|  \| 49 \| 40 or 41 or 42 or 43 or 44 or 45 or 46 or 47 or 48 \| 6911 \| Advanced \| [Display Results](http://ovidsp.dc1.ovid.com/sp-4.02.1a/ovidweb.cgi?&S=CEFBFPHJKLACKGKCKPCKFHHOANPJAA00&SELECT=S.sh%7c&R=49&Process+Action=display)  More \| [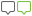](http://ovidsp.dc1.ovid.com/sp-4.02.1a/ovidweb.cgi?&S=CEFBFPHJKLACKGKCKPCKFHHOANPJAA00&R=49&Search+Annotations+Options=SA) \| \|  \| 50 \| Conditioned Medium.mp. \| 25397 \| Advanced \| [Display Results](http://ovidsp.dc1.ovid.com/sp-4.02.1a/ovidweb.cgi?&S=CEFBFPHJKLACKGKCKPCKFHHOANPJAA00&SELECT=S.sh%7c&R=50&Process+Action=display)  More \| [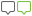](http://ovidsp.dc1.ovid.com/sp-4.02.1a/ovidweb.cgi?&S=CEFBFPHJKLACKGKCKPCKFHHOANPJAA00&R=50&Search+Annotations+Options=SA) \| \|  \| 51 \| Medium, Conditioned.mp. \| 2071 \| Advanced \| [Display Results](http://ovidsp.dc1.ovid.com/sp-4.02.1a/ovidweb.cgi?&S=CEFBFPHJKLACKGKCKPCKFHHOANPJAA00&SELECT=S.sh%7c&R=51&Process+Action=display)  More \| [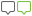](http://ovidsp.dc1.ovid.com/sp-4.02.1a/ovidweb.cgi?&S=CEFBFPHJKLACKGKCKPCKFHHOANPJAA00&R=51&Search+Annotations+Options=SA) \| \|  \| 52 \| Culture Medium, Conditioned.mp. \| 273 \| Advanced \| [Display Results](http://ovidsp.dc1.ovid.com/sp-4.02.1a/ovidweb.cgi?&S=CEFBFPHJKLACKGKCKPCKFHHOANPJAA00&SELECT=S.sh%7c&R=52&Process+Action=display)  More \| [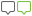](http://ovidsp.dc1.ovid.com/sp-4.02.1a/ovidweb.cgi?&S=CEFBFPHJKLACKGKCKPCKFHHOANPJAA00&R=52&Search+Annotations+Options=SA) \| \|  \| 53 \| Conditioned Culture Media.mp. \| 232 \| Advanced \| [Display Results](http://ovidsp.dc1.ovid.com/sp-4.02.1a/ovidweb.cgi?&S=CEFBFPHJKLACKGKCKPCKFHHOANPJAA00&SELECT=S.sh%7c&R=53&Process+Action=display)  More \| [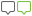](http://ovidsp.dc1.ovid.com/sp-4.02.1a/ovidweb.cgi?&S=CEFBFPHJKLACKGKCKPCKFHHOANPJAA00&R=53&Search+Annotations+Options=SA) \| \|  \| 54 \| Conditioned Media.mp. \| 14699 \| Advanced \| [Display Results](http://ovidsp.dc1.ovid.com/sp-4.02.1a/ovidweb.cgi?&S=CEFBFPHJKLACKGKCKPCKFHHOANPJAA00&SELECT=S.sh%7c&R=54&Process+Action=display)  More \| [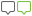](http://ovidsp.dc1.ovid.com/sp-4.02.1a/ovidweb.cgi?&S=CEFBFPHJKLACKGKCKPCKFHHOANPJAA00&R=54&Search+Annotations+Options=SA) \| \|  \| 55 \| Media, Conditioned.mp. \| 1151 \| Advanced \| [Display Results](http://ovidsp.dc1.ovid.com/sp-4.02.1a/ovidweb.cgi?&S=CEFBFPHJKLACKGKCKPCKFHHOANPJAA00&SELECT=S.sh%7c&R=55&Process+Action=display)  More \| [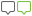](http://ovidsp.dc1.ovid.com/sp-4.02.1a/ovidweb.cgi?&S=CEFBFPHJKLACKGKCKPCKFHHOANPJAA00&R=55&Search+Annotations+Options=SA) \| \|  \| 56 \| Conditioned Culture Medium.mp. \| 378 \| Advanced \| [Display Results](http://ovidsp.dc1.ovid.com/sp-4.02.1a/ovidweb.cgi?&S=CEFBFPHJKLACKGKCKPCKFHHOANPJAA00&SELECT=S.sh%7c&R=56&Process+Action=display)  More \| [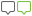](http://ovidsp.dc1.ovid.com/sp-4.02.1a/ovidweb.cgi?&S=CEFBFPHJKLACKGKCKPCKFHHOANPJAA00&R=56&Search+Annotations+Options=SA) \| \|  \| 57 \| 50 or 51 or 52 or 53 or 54 or 55 or 56 \| 38081 \| Advanced \| [Display Results](http://ovidsp.dc1.ovid.com/sp-4.02.1a/ovidweb.cgi?&S=CEFBFPHJKLACKGKCKPCKFHHOANPJAA00&SELECT=S.sh%7c&R=57&Process+Action=display)  More \| [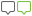](http://ovidsp.dc1.ovid.com/sp-4.02.1a/ovidweb.cgi?&S=CEFBFPHJKLACKGKCKPCKFHHOANPJAA00&R=57&Search+Annotations+Options=SA) \| \|  \| 58 \| 39 or 49 or 57 \| 64223 \| Advanced \| [Display Results](http://ovidsp.dc1.ovid.com/sp-4.02.1a/ovidweb.cgi?&S=CEFBFPHJKLACKGKCKPCKFHHOANPJAA00&SELECT=S.sh%7c&R=58&Process+Action=display)  More \| [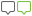](http://ovidsp.dc1.ovid.com/sp-4.02.1a/ovidweb.cgi?&S=CEFBFPHJKLACKGKCKPCKFHHOANPJAA00&R=58&Search+Annotations+Options=SA) \| \|  \| 59 \| Bone Marrow-Derived Macrophages.mp. \| 5517 \| Advanced \| [Display Results](http://ovidsp.dc1.ovid.com/sp-4.02.1a/ovidweb.cgi?&S=CEFBFPHJKLACKGKCKPCKFHHOANPJAA00&SELECT=S.sh%7c&R=59&Process+Action=display)  More \| [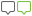](http://ovidsp.dc1.ovid.com/sp-4.02.1a/ovidweb.cgi?&S=CEFBFPHJKLACKGKCKPCKFHHOANPJAA00&R=59&Search+Annotations+Options=SA) \| \|  \| 60 \| Bone Marrow Derived Macrophages.mp. \| 5517 \| Advanced \| [Display Results](http://ovidsp.dc1.ovid.com/sp-4.02.1a/ovidweb.cgi?&S=CEFBFPHJKLACKGKCKPCKFHHOANPJAA00&SELECT=S.sh%7c&R=60&Process+Action=display)  More \| [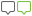](http://ovidsp.dc1.ovid.com/sp-4.02.1a/ovidweb.cgi?&S=CEFBFPHJKLACKGKCKPCKFHHOANPJAA00&R=60&Search+Annotations+Options=SA) \| \|  \| 61 \| Bone Marrow-Derived Macrophage.mp. \| 4383 \| Advanced \| [Display Results](http://ovidsp.dc1.ovid.com/sp-4.02.1a/ovidweb.cgi?&S=CEFBFPHJKLACKGKCKPCKFHHOANPJAA00&SELECT=S.sh%7c&R=61&Process+Action=display)  More \| [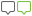](http://ovidsp.dc1.ovid.com/sp-4.02.1a/ovidweb.cgi?&S=CEFBFPHJKLACKGKCKPCKFHHOANPJAA00&R=61&Search+Annotations+Options=SA) \| \|  \| 62 \| Macrophage, Bone Marrow-Derived.mp. \| 1 \| Advanced \| [Display Results](http://ovidsp.dc1.ovid.com/sp-4.02.1a/ovidweb.cgi?&S=CEFBFPHJKLACKGKCKPCKFHHOANPJAA00&SELECT=S.sh%7c&R=62&Process+Action=display)  More \| [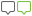](http://ovidsp.dc1.ovid.com/sp-4.02.1a/ovidweb.cgi?&S=CEFBFPHJKLACKGKCKPCKFHHOANPJAA00&R=62&Search+Annotations+Options=SA) \| \|  \| 63 \| Macrophages, Bone Marrow-Derived.mp. \| 57 \| Advanced \| [Display Results](http://ovidsp.dc1.ovid.com/sp-4.02.1a/ovidweb.cgi?&S=CEFBFPHJKLACKGKCKPCKFHHOANPJAA00&SELECT=S.sh%7c&R=63&Process+Action=display)  More \| [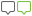](http://ovidsp.dc1.ovid.com/sp-4.02.1a/ovidweb.cgi?&S=CEFBFPHJKLACKGKCKPCKFHHOANPJAA00&R=63&Search+Annotations+Options=SA) \| \|  \| 64 \| Monocyte-Derived Macrophages.mp. \| 6167 \| Advanced \| [Display Results](http://ovidsp.dc1.ovid.com/sp-4.02.1a/ovidweb.cgi?&S=CEFBFPHJKLACKGKCKPCKFHHOANPJAA00&SELECT=S.sh%7c&R=64&Process+Action=display)  More \| [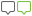](http://ovidsp.dc1.ovid.com/sp-4.02.1a/ovidweb.cgi?&S=CEFBFPHJKLACKGKCKPCKFHHOANPJAA00&R=64&Search+Annotations+Options=SA) \| \|  \| 65 \| Monocyte Derived Macrophages.mp. \| 6167 \| Advanced \| [Display Results](http://ovidsp.dc1.ovid.com/sp-4.02.1a/ovidweb.cgi?&S=CEFBFPHJKLACKGKCKPCKFHHOANPJAA00&SELECT=S.sh%7c&R=65&Process+Action=display)  More \| [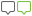](http://ovidsp.dc1.ovid.com/sp-4.02.1a/ovidweb.cgi?&S=CEFBFPHJKLACKGKCKPCKFHHOANPJAA00&R=65&Search+Annotations+Options=SA) \| \|  \| 66 \| Macrophage.mp. \| 362066 \| Advanced \| [Display Results](http://ovidsp.dc1.ovid.com/sp-4.02.1a/ovidweb.cgi?&S=CEFBFPHJKLACKGKCKPCKFHHOANPJAA00&SELECT=S.sh%7c&R=66&Process+Action=display)  More \| [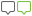](http://ovidsp.dc1.ovid.com/sp-4.02.1a/ovidweb.cgi?&S=CEFBFPHJKLACKGKCKPCKFHHOANPJAA00&R=66&Search+Annotations+Options=SA) \| \|  \| 67 \| Macrophages, Monocyte-Derived.mp. \| 46 \| Advanced \| [Display Results](http://ovidsp.dc1.ovid.com/sp-4.02.1a/ovidweb.cgi?&S=CEFBFPHJKLACKGKCKPCKFHHOANPJAA00&SELECT=S.sh%7c&R=67&Process+Action=display)  More \| [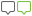](http://ovidsp.dc1.ovid.com/sp-4.02.1a/ovidweb.cgi?&S=CEFBFPHJKLACKGKCKPCKFHHOANPJAA00&R=67&Search+Annotations+Options=SA) \| \|  \| 68 \| Macrophage, Monocyte-Derived.mp. \| 15 \| Advanced \| [Display Results](http://ovidsp.dc1.ovid.com/sp-4.02.1a/ovidweb.cgi?&S=CEFBFPHJKLACKGKCKPCKFHHOANPJAA00&SELECT=S.sh%7c&R=68&Process+Action=display)  More \| [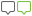](http://ovidsp.dc1.ovid.com/sp-4.02.1a/ovidweb.cgi?&S=CEFBFPHJKLACKGKCKPCKFHHOANPJAA00&R=68&Search+Annotations+Options=SA) \| \|  \| 69 \| Macrophages, Monocyte Derived.mp. \| 46 \| Advanced \| [Display Results](http://ovidsp.dc1.ovid.com/sp-4.02.1a/ovidweb.cgi?&S=CEFBFPHJKLACKGKCKPCKFHHOANPJAA00&SELECT=S.sh%7c&R=69&Process+Action=display)  More \| [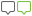](http://ovidsp.dc1.ovid.com/sp-4.02.1a/ovidweb.cgi?&S=CEFBFPHJKLACKGKCKPCKFHHOANPJAA00&R=69&Search+Annotations+Options=SA) \| \|  \| 70 \| Monocyte-Derived Macrophage.mp. \| 747 \| Advanced \| [Display Results](http://ovidsp.dc1.ovid.com/sp-4.02.1a/ovidweb.cgi?&S=CEFBFPHJKLACKGKCKPCKFHHOANPJAA00&SELECT=S.sh%7c&R=70&Process+Action=display)  More \| [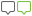](http://ovidsp.dc1.ovid.com/sp-4.02.1a/ovidweb.cgi?&S=CEFBFPHJKLACKGKCKPCKFHHOANPJAA00&R=70&Search+Annotations+Options=SA) \| \|  \| 71 \| 59 or 60 or 61 or 62 or 63 or 64 or 65 or 66 or 67 or 68 or 69 or 70 \| 362731 \| Advanced \| [Display Results](http://ovidsp.dc1.ovid.com/sp-4.02.1a/ovidweb.cgi?&S=CEFBFPHJKLACKGKCKPCKFHHOANPJAA00&SELECT=S.sh%7c&R=71&Process+Action=display)  More \| [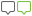](http://ovidsp.dc1.ovid.com/sp-4.02.1a/ovidweb.cgi?&S=CEFBFPHJKLACKGKCKPCKFHHOANPJAA00&R=71&Search+Annotations+Options=SA) \| \|  \| 72 \| 34 and 58 and 71 \| 424 \| Advanced \| [Display Results](http://ovidsp.dc1.ovid.com/sp-4.02.1a/ovidweb.cgi?&S=CEFBFPHJKLACKGKCKPCKFHHOANPJAA00&SELECT=S.sh%7c&R=72&Process+Action=display)  More \|  \| |
| --- | --- | --- | --- | --- | --- | --- | --- | --- | --- | --- | --- | --- | --- | --- | --- | --- | --- | --- | --- | --- | --- | --- | --- | --- | --- | --- | --- | --- | --- | --- | --- | --- | --- | --- | --- | --- | --- | --- | --- | --- | --- | --- | --- | --- | --- | --- | --- | --- | --- | --- | --- | --- | --- | --- | --- | --- | --- | --- | --- | --- | --- | --- | --- | --- | --- | --- | --- | --- | --- | --- | --- | --- | --- | --- | --- | --- | --- | --- | --- | --- | --- | --- | --- | --- | --- | --- | --- | --- | --- | --- | --- | --- | --- | --- | --- | --- | --- | --- | --- | --- | --- | --- | --- | --- | --- | --- | --- | --- | --- | --- | --- | --- | --- | --- | --- | --- | --- | --- | --- | --- | --- | --- | --- | --- | --- | --- | --- | --- | --- | --- | --- | --- | --- | --- | --- | --- | --- | --- | --- | --- | --- | --- | --- | --- | --- | --- | --- | --- | --- | --- | --- | --- | --- | --- | --- | --- | --- | --- | --- | --- | --- | --- | --- | --- | --- | --- | --- | --- | --- | --- | --- | --- | --- | --- | --- | --- | --- | --- | --- | --- | --- | --- | --- | --- | --- | --- | --- | --- | --- | --- | --- | --- | --- | --- | --- | --- | --- | --- | --- | --- | --- | --- | --- | --- | --- | --- | --- | --- | --- | --- | --- | --- | --- | --- | --- | --- | --- | --- | --- | --- | --- | --- | --- | --- | --- | --- | --- | --- | --- | --- | --- | --- | --- | --- | --- | --- | --- | --- | --- | --- | --- | --- | --- | --- | --- | --- | --- | --- | --- | --- | --- | --- | --- | --- | --- | --- | --- | --- | --- | --- | --- | --- | --- | --- | --- | --- | --- | --- | --- | --- | --- | --- | --- | --- | --- | --- | --- | --- | --- | --- | --- | --- | --- | --- | --- | --- | --- | --- | --- | --- | --- | --- | --- | --- | --- | --- | --- | --- | --- | --- | --- | --- | --- | --- | --- | --- | --- | --- | --- | --- | --- | --- | --- | --- | --- | --- | --- | --- | --- | --- | --- | --- | --- | --- | --- | --- | --- | --- | --- | --- | --- | --- | --- | --- | --- | --- | --- | --- | --- | --- | --- | --- | --- | --- | --- | --- | --- | --- | --- | --- | --- | --- | --- | --- | --- | --- | --- | --- | --- | --- | --- | --- | --- | --- | --- | --- | --- | --- | --- | --- | --- | --- | --- | --- | --- | --- | --- | --- | --- | --- | --- | --- | --- | --- | --- | --- | --- | --- | --- | --- | --- | --- | --- | --- | --- | --- | --- | --- | --- | --- | --- | --- | --- | --- | --- | --- | --- | --- | --- | --- | --- | --- | --- | --- | --- | --- | --- | --- | --- | --- | --- | --- | --- | --- | --- | --- | --- | --- | --- | --- | --- | --- | --- | --- | --- | --- | --- | --- | --- | --- | --- | --- | --- | --- | --- | --- | --- | --- | --- | --- | --- | --- | --- | --- | --- | --- | --- | --- | --- | --- | --- | --- | --- | --- | --- | --- | --- | --- | --- | --- | --- | --- | --- | --- | --- | --- | --- | --- | --- | --- | --- | --- | --- | --- | --- | --- | --- | --- | --- | --- | --- | --- | --- | --- | --- | --- | --- | --- | --- | --- | --- | --- | --- | --- | --- | --- | --- | --- | --- | --- | --- | --- | --- | --- | --- | --- | --- | --- | --- | --- | --- | --- | --- | --- | --- |

Appendix 4: Methodological quality assessment document (the number of ‘yes’ answers was counted for each study to give a total score out of 8)

| Section and topic | No. | Quality criteria | Yes | No |
| --- | --- | --- | --- | --- |
| Title / Keywords / Introduction | 1 | Were the study hypothesis/aim/objective clearly described |  |  |
| Method | 2 | Were the animal model for the study well-described |  |  |
|  | 3 | Were the method well-described |  |  |
|  | 4 | Were the data collected time point clearly defined |  |  |
|  | 5 | Were the main outcome measures clearly defined |  |  |
|  | 6 | Were the experiment group well compared with the control group |  |  |
| Discussion | 7 | Were the results well-described |  |  |
|  | 8 | Were the articles discussed the limitation |  |  |

Kathleen Wells, Julia H. Littell. Study Quality Assessment in Systematic Reviews of Research on Intervention Effects. Research on Social Work Practice. 2009;19: 52-62.

Appendix 5: Summary of selected studies and methodological score

| Study | 1 | 2 | 3 | 4 | 5 | 6 | 7 | 8 | Quality score |
| --- | --- | --- | --- | --- | --- | --- | --- | --- | --- |
| Chang J. et al | No | Yes | Yes | Yes | No | No | Yes | Yes | 5 |
| Cosenza S. et al | Yes | Yes | Yes | Yes | Yes | No | Yes | Yes | 7 |
| Sicco C. et al | No | Yes | Yes | Yes | Yes | Yes | Yes | Yes | 7 |
| Hyvärinen K. et al | Yes | Yes | Yes | Yes | Yes | No | No | Yes | 6 |
| Zhang S. et al | Yes | Yes | Yes | Yes | Yes | Yes | Yes | No | 7 |
| Chamberlain CS. et al | Yes | Yes | Yes | Yes | Yes | Yes | Yes | Yes | 8 |
| Pacienza N. et al | Yes | Yes | Yes | Yes | Yes | No | No | No | 5 |
| Shen H. et al | Yes | Yes | Yes | Yes | Yes | Yes | Yes | No | 7 |
| Shi Z. et al | Yes | Yes | Yes | Yes | Yes | Yes | Yes | No | 7 |
| Li Y. et al | Yes | Yes | Yes | Yes | Yes | Yes | No | Yes | 7 |
